# Untargeted Metabolomics of Extracts from Faecal Samples Demonstrates Distinct Differences between Paediatric Crohn’s Disease Patients and Healthy Controls but No Significant Changes Resulting from Exclusive Enteral Nutrition Treatment

**DOI:** 10.3390/metabo8040082

**Published:** 2018-11-22

**Authors:** Adel Alghamdi, Konstantinos Gerasimidis, Gavin Blackburn, Didem Akinci, Christine Edwards, Richard K. Russell, David G. Watson

**Affiliations:** 1 Strathclyde Institute of Pharmacy and Biomedical Sciences, University of Strathclyde, Glasgow G4 0RE, UK; adel.alghamdi@strath.ac.uk; 2 School of Medicine, College of MVLS, University of Glasgow, Glasgow G31 2ER, UK; 3 Glasgow Polyomics, Translational Cancer Research Centre, University of Glasgow Garscube Campus, Glasgow G61 1QH, UK; Gavin.Blackburn@glasgow.ac.uk; 4 Department of Paediatric Gastroenterology, Hepatology and Nutrition, Royal Hospital for Children, Glasgow G51 4TF, UK; didem-akinci@hotmail.com (D.A.); Christine.Edwards@glasgow.ac.uk (C.E.); richardrussell@nhs.net (R.K.R.)

**Keywords:** Crohn’s disease, EEN, Metabolomics, LC-MS, multivariate analysis

## Abstract

Metabolomic profiling using high resolution mass spectrometry with hydrophilic interaction chromatography was applied to 11 faecal extracts from eleven healthy children and to 43 faecal extracts from eleven children undergoing exclusive enteral nutrition for the treatment of active Crohn’s disease (CD) at timepoints before, during (15, 30, and 60 days), and after treatment. Differences between the control and CD samples were identified at each timepoint. An orthogonal partial least square-discriminant analysis (OPLS-DA) model identified eight metabolites that were normally distributed according to Q-Q plots. The OPLS-DA model was able to discriminate the CD samples from the controls at every timepoint, but the model was not able to differentiate the CD samples from one another at the different timepoints during treatment with exclusive enteral nutrition. The differentiated metabolites identified in the CD samples included tyrosine, an ornithine isomer, arachidonic acid, eicosatrienoic acid, docosatetraenoic acid, a sphingomyelin, a ceramide, and dimethylsphinganine. Despite successful treatment, underlying differences remained in the metabolome of the CD patients. These differences dominated the separation of the samples when multivariate methods were applied.

## 1. Introduction

Crohn’s disease (CD) is a component of Inflammatory Bowel Disease (IBD), a multifactorial disorder likely resulting from altered immune responses to commensal or pathogenic gut microbes under the influence of an environmental factor, including diet [1]. Children and adolescents represent 15 to 20% of all CD cases, in whom the disease presents more extensively and severely [2]. The disease has distinct stages: onset, severity, progression, remission, and relapse. A dysbiotic gut microbiota is thought to play a role in the disease pathogenesis. Correlations between CD and diet are believed to be equally important, but the specific molecular interactions remain unclear. Therefore, knowledge of a defined metabolomic fingerprint in CD could be useful for diagnosis, treatment, detection of disease pathogenesis, and prediction of disease progression.

Exclusive Enteral Nutrition (EEN) is the most common treatment for paediatric CD in the UK and the rest of Europe [3]. EEN is a liquid-only diet comprised of a proprietary nutritional feed that is administered to CD patients for up to eight weeks. EEN induces clinical remission in approximately 80% of cases [4] and results in mucosal healing more often than treatment with high doses of oral steroids [5]. Two mechanisms have been suggested for the effectiveness of EEN treatment. The first relates to changes in the gut microbiota composition and metabolism [6,7]. The second involves exclusion of dietary triggers of the disease, such as food emulsifiers and preservatives [8]. However, the exact mechanism of EEN treatment has not been fully elucidated and requires further investigation.

Metabolomics is an indispensable research tool for the identification and tracking of biomarkers in biological systems and fluids. This holistic approach provides the broadest array of functional information in systems biology [9]. An unbiased, data-driven method, metabolomics presents a novel means of interrogating biological systems that could lead to new hypotheses and biological knowledge. In a typical metabolomics study, complex extracts or body fluids are analysed and compared by various methods to generate metabolic fingerprints [10]. The primary metabolomic techniques are either based on nuclear magnetic resonance (NMR) [9] or mass spectrometry (MS) [10]. When MS is applied, it is often used in combination with gas chromatography (GC-MS) or liquid chromatography (LC-MS). Due to the wide structural and chemical diversity of metabolites, a single analytical method may not provide a complete index of all the metabolites present in an organism at the time the sample was obtained [11]. Consequently, a combination of methods is preferred for metabolomic studies. The recorded dataset is processed and compared to a range of metabolic fingerprints using multivariate data analysis (MVDA). This analysis can reveal features in the dataset that could be linked to biomarkers for differential diagnosis and monitoring of treatment [12]. There have been a number of previous studies which have applied metabolomics profiling in IBD without any firm agreement with regard to the biomarkers indicative of the disease [1,13,14,15,16,17]. Few studies have applied LC-MS to the analysis of faecal extracts and the majority of studies have used NMR or GC-MS for the analysis [16,17]. There are also no studies in children with CD during treatment with EEN in comparison with healthy controls. Comparing differences between healthy controls and CD patients over the course of treatment offers the opportunity to unravel factors implicated in disease pathogenesis and the mechanism of EEN action.

In the current study, metabolomic profiling based on high resolution LC-MS data was used to identify significantly differentiated metabolites in the faecal samples of children with CD before, during, and after EEN treatment. The relative abundances of these identified metabolites were examined and compared to the metabolomic profiles of healthy controls.

## 2. Results

### 2.1. Pooled Samples

**Figure 1:**
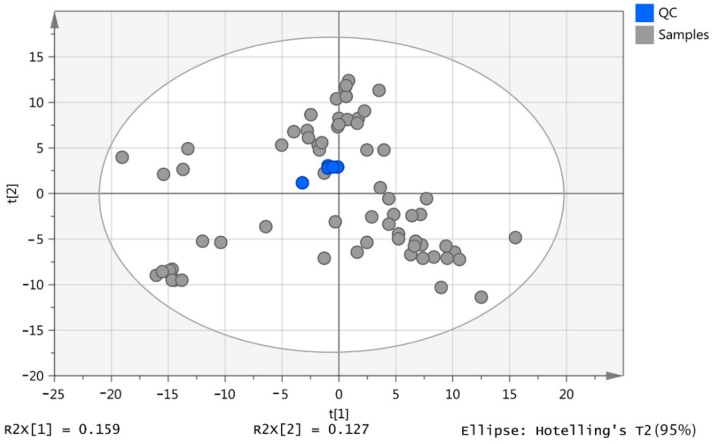
2D Scores plot of the principal components analysis (PCA) for the quality control (QC) samples (blue) and all samples (grey) based on 606 putative metabolites.

The initial screening detected 606 putatively identified metabolites. The pooled samples (*n* = 5) were clustered, indicating that no technical errors occurred during the analysis (Figure 1). Metabolites were identified to Metabolomic Standard Initiative (MSI) levels 1 or 2, and matching was carried out against authentic standards [18] where available. The details of our standard mixtures were provided in a previous publication [19]. To quantify the precision of the determinations, the relative standard deviation (RSD) was calculated between the five pooled samples based on the total intensities in each sample, resulting in an RSD of 14%. Using the percentage RSD criteria, metabolites with an RSD > 30% were excluded, accounting for 230 compounds. The remaining 376 metabolites were retained in the study, and the analysis continued as described below.

### 2.2. Data Visualisation

As shown in Figure S1a,b, log2 transformation improved data clustering and separation. The samples from the children in the healthy control (HC) group were clearly separated from the CD patient groups. During EEN treatment, the serially collected samples PB, PC, and PD clustered together on the left half of the ellipse. After the CD patients completed EEN treatment and returned to their free habitual diet (samples PE), the samples appeared between the pre-treatment and healthy control groups. There was a clear separation between the pre-treatment (PA) and the HC groups.

An orthogonal partial least square-discriminant analysis (OPLS-DA) model was constructed, and the validation process was carried and the data are shown in Table 1 for models based on 376 metabolites. The only valid models were PA vs. HC and PA vs. PC. Both models produced a goodness of prediction (Q2) > 0.5, and the differences between the goodness of fit (R2) and Q2 were less than 0.3. However, as shown in Table 2, the HC group in comparison with all groups produced valid and significant models.

**Figure 2:**
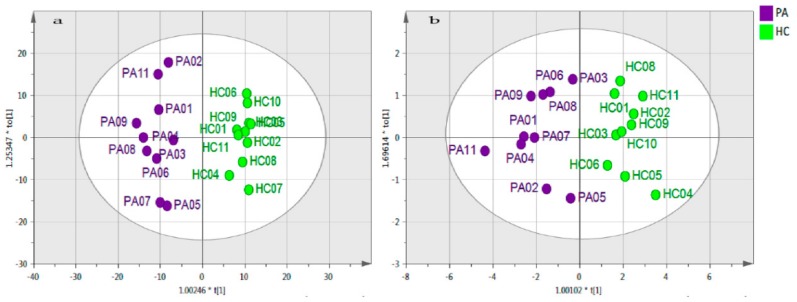
OPLS-DA score plot of pre-EEN samples (PA) against healthy controls (HC). (**a**) The OPLS-DA model based on 376 metabolites. The model consists of one predictive *x*-score component, component t[1], and one orthogonal *x*-score component to[1]. The t[1] component explains 19.1% of the predictive variation in *x*, while the to[1] component explains 11.6% of the orthogonal variation in *x*, R2X (cum) =  0.307. The goodness of fit (R2) = 0.96, the goodness of prediction (Q2) = 0.848 and the *p* value associated with the cross-validation analysis of variance (CV-ANOVA) = 5.41 × 10^−6^. The PA10 sample was excluded as outlier. (**b**) The OPLS-DA model based on 8 differentiated metabolites. The model consists of one predictive *x*-score component, component t[1], and one orthogonal *x*-score component, to[1]. The t[1] component explains 677% of the predictive variation in *x*, while the to[1] component explains 11.7% of the orthogonal variation in *x*, R2X (cum) = 0307. R2 = 0.837, Q2 = 0.57, and *p* CV-ANOVA = 1.47 × 10^−2^. The PA10 and HC07 samples were excluded as outliers. Both models were based on log base 2 variables that were Pareto scaled.

By applying the methodology described in Supplementary Figure S2, eight differentiated metabolites were identified in the PA and HC samples (Table 3). There was a clear separation between these groups (Figure 2a). The final model remained valid after data analysis, even for the short list of metabolites as shown in Figure 2b.

**Figure 3:**
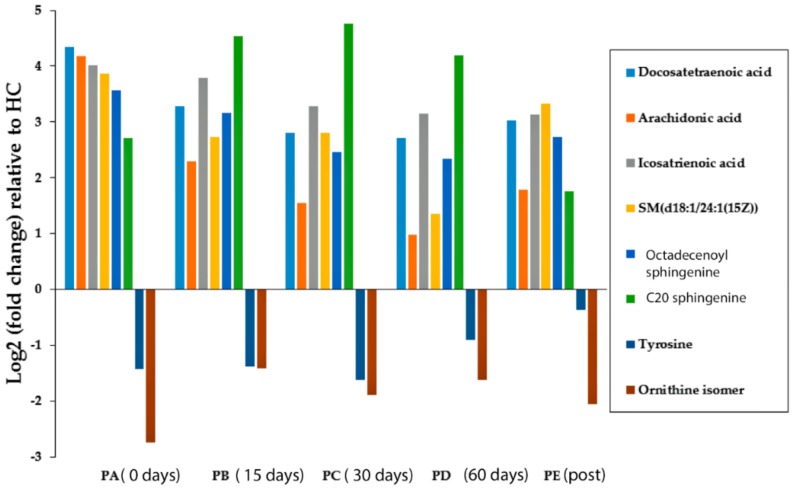
Log2 of the fold-change in the eight differentiated metabolites in the CD groups (before, during, and after EEN treatment) compared with the group of healthy controls. (HC) Healthy control children, (PA) CD children pre-EEN treatment, (PB) CD children 15 days during EEN treatment, (PC) CD children 30 days during EEN treatment, (PD) CD children 60 days during EEN treatment, (PE) CD children back to a free diet.

The levels of an ornithine isomer and tyrosine were significantly lower in the PA samples than the HC group (Log2 (PA/HC) = −2.74 and −1.43 for the ornithine isomer and tyrosine, respectively). The remaining metabolites were found in a higher abundance in children with active CD at the sampling points compared to the HC group (Figure 3). The eight marker compounds remained largely significantly lower or higher than the controls although some of the metabolites moved closer to the control levels, with the effect being most marked for arachidonic acid and ceramide. The retention times of four of the maker compounds could be matched against available standards. Thus, four of the compounds were only identified to MSI level 2 [16]. MS^n^ fragmentation was carried out using an Orbitrap Fusion for these compounds with mixed success. The details of the characterization of the compounds are given in Table 4. Quite definitive identification of the C20 sphinganine and the C18 sphingosine was achieved. Clear and logical fragments were obtained for the isomer of ornithine although the MS^2^ was weak, however it would be difficult to propose a definitive structure based on these. The ceramide yielded abundant fragments it was not possible to make sense of these. There was one fragment at 264.2 in low resolution in MS^3^ mode which was the same as a fragment obtained for the C18 sphingosine which is associated with the C18 sphinganine core of the molecule. Correlation plots for the marker compounds against the values obtained for calprotectin for the samples did not reveal any strong correlation between the peak areas for the marker metabolites and the calprotectin values.

The Supplementary Table S1 shows a complete list of significant metabolites in ascending molecular weight, indicating where the retention time of the metabolite was matched to that of a standard as well as the *p* values and ratios obtained for the comparison of the HC group against the pre-treatment samples.

The fatty acids identified by the ZICpHILIC screen were not strongly retained on the column. To confirm their identity, two marker fatty acids, arachidonic acid and eicosatrienoic acid, were matched against the retention times of their corresponding standards on a C4 reversed phase column. A quantitative estimate of the fatty acids in the samples was performed by preparing calibration curves in the range 0.1 µg to 16 µg/ml and estimating the fatty acid content in the faecal extracts for the HC and pre-treatment samples based on the calibration lines. Table 5 reports the levels of the fatty acids in the HC and pre-EEN treatment samples in µg/g.

## 3. Discussion

In this study, several amino acids and amino acid metabolites were present at significantly higher levels in the pre-EEN treatment samples of the CD patients in comparison with controls. These observations are generally in line with Kolho et al. who found elevations of the following metabolites in faecal samples from CD patients: aspartate, glycine, tryptophan, carnosine, allantoin, citrulline, serine, threonine, ornithine, creatine, asparagine, choline, kynurenine, histidine, taurine, phenylalanine, alanine, and metanephrine [13]. In their study, these elevated metabolites could be used to discriminate between the CD patients and the healthy controls. Jannson et al. found that tyrosine and its metabolites as well as phenylalanine and tryptophan were significantly higher in CD patients [1]. In the current study, tyrosine levels were significantly lower in the pre-EEN treatment versus the HC samples and remained either lower or significantly lower throughout the treatment and post-treatment samples (Table S1 and Figure S4).

In another study, Bjerrum et al. found that leucine, isoleucine, valine, lysine, alanine, tyrosine, phenylalanine, and glycine were all present at high levels in faecal extracts from CD patients compared to healthy controls [14]. The study by Bjerrum is in agreement with our results except for the tyrosine levels, which were consistently lower. Schicho et al. reported increased levels of methionine, lysine, glycine, arginine, and proline and decreased levels of valine, tyrosine, and serine in faecal extracts from CD patients [15]. Schicho et al.’s findings regarding tyrosine levels reflect our tyrosine results, but we found that valine and serine were either consistently higher than the controls or no different from the controls.

In the current study, we used a rigorous selection procedure to determine important markers that could discriminate between HC and pre-treatment CD samples and then follow these markers during the course of EEN. Given the small set of patients, it was not possible to assume that the peak areas obtained for the metabolites were normally distributed, even after logarithmic transformation. Although *p* values have been reported in previous studies using similarly small sample sets, we could not be certain that a null hypothesis could be rejected without conducting a Q-Q test. For example, in the current study, taurine is significantly higher in most of the treated and untreated patient samples in comparison with the control (Table S1), and it is tempting to conclude that taurine is an important disease marker, given its anti-inflammatory effects [20]. However, the Q-Q test indicated that taurine was not normally distributed and appears to be normally distributed in two groups (Figure S3); thus, its *p* values could not be reported. The same was true for acetyl choline, which was significantly higher in all the patient samples but did not pass the QQ test returning a low R^2^ value (Figure S3).

Q-Q tests are time consuming to perform, and it is not possible to carry these out for large numbers of markers. Multivariate statistics using the SIMCA-P software (14.1) was applied to solve this problem. The multivariate models produced by the SIMCA-P software do not assume a normal distribution of marker compounds. In the model shown in Figure 5, the non-parametric jack-knife test [21] was used to select reliable markers, reducing the marker list to eight. A Q-Q test was then applied to these markers to check for normal distribution. Six out of the eight markers were normally distributed with the ceramide (SM (d18:1/24:1)) having too many missing values to give normal distribution (Figure S3).

Large differences were identified in the levels of these marker compounds between the HC and the CD patients. Only two marker compounds were reduced in the CD patients, tyrosine and an ornithine isomer. Tyrosine has previously been reported as a CD marker that was increased in faecal extracts from CD patients [13] and decreased in the plasma from CD patients. In our study, the low tyrosine levels were not significantly changed after EEN treatment in comparison with the HC group. Several tyrosine metabolites were also present in low amounts in the CD patients, including dopamine, noradrenaline, metanephrine, normetanephrine, adrenaline, and DOPA. Catecholamines are normally at very low concentrations in plasma, but the levels excreted in urine are generally much higher. There is no substantial literature on the levels of catecholamines in faeces. Further research is needed on this issue, as it was not possible to validate the identities of these putatively identified markers when their retention times were compared with authentic standards.

The other marker compound that was found at reduced levels in our analysis of CD patients, with an average intensity of 0.15 compared to that in the healthy controls, was an ornithine isomer. Since ornithine has two basic centres, it runs very late in our HILIC method, while the marker compound ran much earlier than the ornithine standard. Two ornithine isomers were present in our database; one of these would have been expected to elute late from the column since it is a diamine, but N4-acetyl-N4-hydroxy-1-aminopropane would be expected to elute early. This ornithine isomer is found as a biosynthetic intermediate in the synthesis of siderophores in Rhizobia bacteria, but whether similar pathways might exist in the microbiome bacteria is not known [22].

Dietary omega 6 fatty acids that include arachidonic acid and eicosatrienoic acid may be implicated in IBD [23]. In our study, the levels of arachidonic acid, eicosatrienoic acid, and docosatetraenoic acid were much higher in the CD patients compared to the HC group (Table 2 and Table 4) and remained high both pre- and post-EEN treatment. These fatty acids cannot have derived from the enteral nutrition formula since their levels were higher in both the PA and PE samples compared with the HC group. In addition, Table 2 indicates that elevation does not occur for most of the fatty acids evaluated in this study. The greatest accumulations were seen for three C20 polyunsaturated acids and a C22 polyunsaturated acid. In contrast, there was not much difference in the levels of C16 and C18 acids between the CD patients at all the time points and the HC group. These results suggest that CD pathogenesis or progression might be related to the metabolism or absorption of this fatty acid class and replicate findings of other groups that demonstrate higher levels too [24]. Although these fatty acids are not strongly indicative of the effectiveness of treatment. It can be seen from the data in Table 4 that while the fatty acid marker compounds are much higher in the CD group than in the HC group, there is a wide variation of levels within the CD group, this might give an indication of the severity of the disease but since the calprotectin measurements, as mentioned above, did not correlate with the levels of the fatty acids in the samples there is no means of confirming this.

Omega 6 fatty acids have been shown to be pro-inflammatory in a mouse model [25]; those pro-inflammatory effects were suppressed in transgenic mice that were capable of converting omega 6 to omega 3 fatty acids. Omega 3 fatty acids have been shown to promote the formation of intestinal alkaline phosphatase, which breaks down the potent pro-inflammatory lipopolysaccharides produced by *Escherichia coli*, which may, in turn, drive CD inflammation. In our study, EEN treatment had some impact on the levels of these fatty acids, but they still remained higher in the CD patients than in the controls throughout all phases of treatment.

The role of sphingomyelins and ceramides in CD has been investigated, with variable findings [26,27,28]. In the current study, three of the elevated markers in the CD patients were in the sphingolipid category. The sphingolipid levels were not greatly affected by EEN treatment. A previous study observed that probiotic bacteria in a mouse IBD model produced a neutral sphingomyelinase that could convert sphingomyelin into ceramides, promoting apoptosis of mucosal immune cells leading to improved homeostasis and reduced inflammation [24]. This theory would explain the elevated sphingomyelin levels in the current study, but it does not conform to the elevated levels of pro-apoptotic ceramides found in the CD samples (Supplementary Table S3). Of note, Sewell et al. found no differences in the ceramide composition of macrophages taken from CD patients compared to a control group. The ceramides monitored in that study corresponded largely to those shown in Supplementary Table S2 [28].

The partial elucidation of the structures of the marker compounds for which matching standards were not available was carried out and is summarised in Table 4. Confidence in the identity of two of the sphingolipids is high and comprehensive fragmentation schemes are shown in Figures S6 and S10. However, complete elucidation of the structure of the ceramide so far eludes us.

## 4. Materials and Methods

### 4.1. Chemicals and Solvents

High-performance liquid chromatography (HPLC) grade acetonitrile (ACN) was purchased from Fisher Scientific (Loughborough, UK), and HPLC grade water was produced by a Direct-Q3 UltrapureWater System (Millipore, Watford, UK). AnalaR-grade formic acid (98%) was obtained from BDH-Merck (Poole, UK). Authentic stock standard metabolites (Sigma-Aldrich, Poole, UK) were prepared as previously described [29] and diluted four times with ACN.

The quantification of fatty acids was performed using commercial standards: arachidonic acid, (CAS number 506-32-1, Sigma-Aldrich, Poole, UK) and Cis-8, 11, 14-Eicosatrienoic acid (CAS number 1783-84-2, Sigma-Aldrich, Poole, UK). All other standards were obtained from Sigma Aldrich, Poole, UK.

### 4.2. Samples and Sample Preparation

This study received ethics approval by the Yorkhill Research Ethics Committee (05/S0708/66). Both carers and patients provided written consent. Serial faecal samples were collected during exclusive enteral nutrition (*n* = 54) from 11 CD children (4 females, age mean (SD): 11.5 (2.4)) (Table 6). A single spot sample was collected for comparative purposes from 11 age and gender matched healthy controls (4 females, age mean (SD): 10.2 (2.3)) with no familiar history of IBD. From the 11 children with CD, 7 were newly diagnosed, treatment naïve and four received a repeat course of EEN (all within a year of diagnosis) due to disease relapse. All patients completed a 7–8 weeks course of exclusive enteral using Modulen IBD (Nestle, Vevey, Switzerland). Four patients (2 newly diagnosed and 2 patients on relapse) were on concomitant treatment with azathioprine and three on 5-aminoasalicylates. No patient had received antibiotics within 3 months prior to recruitment. At treatment initiation, the mean (SEM) BMI *z*-score was ‒1.61 (0.27) (BMI 13.8 ± 1.4) with 7 out of 11 (64%) patients classified as undernourished (BMI < 2nd centile). Following 4- and 8-week treatment on EEN, the baseline BMI *z*-score significantly (both *p* < 0.001) increased by 1.6 (0.38) (BMI 15.7 ± 1.3) and 1.7 (0.35) SD (BMI 16.2 ± 1.5) respectively (this data is summarised in Table 7). Seven patients had a BMI *z*-score below the 2nd centile at treatment initiation, all patients had active disease (Paediatric Disease Activity Index (PCDAI) > 10 units). At treatment completion, 7 patients entered in clinical remission (PCDAI < 10 units); 3 others had a significant improvement in clinical disease activity but did not enter clinical remission (PCDAI > 10 units) and one patient did not respond to treatment and oral steroid was initiated following EEN cessation at 8 weeks.

From the children with CD, samples were collected starting either before EEN initiation or the first sample passed after EEN initiation to a maximum of five days after EEN initiation (PA). Follow up samples were collected during treatment at 15 days after EEN initiation (PB), 30 days after EEN initiation (PC), and 60 days after EEN initiation (PD). A final sample (PE) was collected two to four months post treatment after the patients had resumed their free diet. Faecal calprotectin (FC, mg/kg) was raised in all patients prior to EEN initiation (median, IQR: 2262, 2089:2582) and significantly decreased after 30 [FC change (SEM) from treatment initiation at 15 days: −483 (211), *p* = 0.123; at 30 days: −679 (204), *p* = 0.012; at 60 days: −1002 (211), *p* < 0.001]. 4 out of the 11 patients had a FC below 150 mg/kg at the end of EEN. FC concentration returned to pre-treatment levels within 2–4 months of food reintroduction (median, IQR, min-max: 2248, 1969–2431, 1632–2495).

All samples were freeze dried then extracted immediately with chloroform/methanol/water (1:3:1 *v*/*v*). The extracts were stored at −80 °C until analysis by LC-MS. Calprotect values were determined as described previously [30] and are shown in Table S3. Samples were randomized to avoid inter-batch differences. Pooled samples (*n* = 5) were prepared from a combination of all samples and intermittently injected throughout the sequence. The samples were randomised and analysed in batches of 13 faecal extracts with one pooled sample in between batches in LC-MS analysis.

### 4.3. LC-MS Analysis

Mobile phase solvents were freshly prepared and stored at room temperature for up to 48 h. Mobile phase A: ammonium carbonate buffer (20 mM, pH 9.2) was prepared by the addition of 1.92 g of ammonium carbonate to 800 mL of HPLC-grade water, followed by an adjustment to pH 9.2 with ammonia solution and then filled to a volume of 1 L. Mobile phase B: HPLC-grade acetonitrile only.

The metabolites were eluted from the ZICpHILIC column (150 × 4.6 mm, 5 µm particle size) supplied by Hichrom Ltd. (Reading, UK) with a mobile phase consisting of 20 mM ammonium carbonate in HPLC-grade water (solvent A) and acetonitrile (solvent B), at a flow rate of 0.3 mL/min. The elution gradient was an A:B ratio of 20:80 at 0 min, 80:20 at 30 min, 92:8 at 30 min 92:8 at 35 min, 20:80 at 36 min, and 20:80 at 45 min.

An ACE C4 column was used to estimate the unsaturated fatty acids. The mobile phase for the elution of the ACE C4 column consisted of 1 mM acetic acid in water (A) and 1 mM acetic acid in acetonitrile (B) at a flow rate of 0.4 mL/min. The elution gradient was as follows: A:B ratio 60:40 at 0 min, 0:100 at 30 min, 0:100 at 36 min, 60:40 at 37 min, and 60:40 at 41 min.

The nitrogen sheath and auxiliary gas flow rates were maintained at 50 and 17 arbitrary units. The electrospray ionisation (ESI) interface was operated in both positive and negative modes. The spray voltage was 4.5 kV for the positive mode and 4.0 kV for negative mode, while the ion transfer capillary temperature was 275 °C. Full scan data was obtained in the mass-to-charge range of m/z 75 to m/z 1200 for both ionisation modes. The MS system was fully calibrated prior to running the samples according to the manufacturer’s guidelines. The resulting data was acquired using the XCalibur 2.1.0 software package (Thermo Fisher Scientific, Bremen, Germany). Additional experiments were carried out on an Orbitrap Fusion connected with a ZICpHILIC column using the conditions above. The nitrogen sheath and auxiliary gas flow rates were maintained at 40 and 5 arbitrary units. ESI interface was operated positive mode at 4.3 kV, the ion transfer capillary temperature was 325 °C. MS^2^ and MS^3^ spectra were obtained using a collision energy of 30 V. For data dependent MS^n^ experiments the inclusion list consisted of the ions at m/z 133.097, 328.32, 564.53, and 813.68.

### 4.4. Data Pre-processing and Modelling

The data was extracted by using MZ Match software (version 1, http://mzmatch.sourceforge.net/) [31], and the identification of putative metabolites was made via the macro-enabled Excel file, IDEOM (http://mzmatch.sourceforge.net/ideom.html) [32]. The lists of the metabolites obtained from these searches were then manually evaluated by considering the quality of their peaks and their retention time match with the standard metabolite mixtures run in the same sequence. All reported metabolites were within 3 ppm of their exact masses.

The Excel sheet output provided from Mzmatch was pre-processed to improve data quality. The RSD ((standard deviation/mean) × 100)) for each of the metabolites was calculated using quality control (QC) samples (*n* = 5), and the metabolites were excluded from the analysis if the RSD was > 30%. Metabolites were also excluded if the missing values were more than 20% in the biological samples. The remaining metabolites were then transformed using log base 2 to reduce data skewing and improve data normality [33]. The multivariate analysis and data mining were carried out using SIMCA-P software v.14.1 (MKS Umetrics AB, Umeå Sweden). The data were Pareto scaled, which divided each metabolite intensity by the square root of its standard deviation [30]. Then, unsupervised principal components analysis (PCA) was used to evaluate the QC samples and exclude technical errors.

After the data was transformed and Pareto scaled, the groups were defined, and a supervised OPLS-DA model was applied to all metabolites. In this model, the variation was divided into two analyses. The first was a prediction variation, which is the correlated variation between X and Y. This variation represents the inter group variation. The second analysis was an orthogonal variation, which is orthogonal to the first analysis and the uncorrelated variation between *X* and *Y*. This variation analysis represents the intra group variation [34].

### 4.6. Model Validation

The next step was to evaluate the separation between the groups and to start the group comparisons (Table 2). The model parameters cumulated the amount of variation in matrix X R2X (cum), R2, and Q2, and a permutation test was examined to evaluate the model’s validity. The significant differences in the model were assessed by calculating the *p*-values from the cross-validation analysis of variance (CV-ANOVA). A *p* value of 0.05 was used as the significant value. The difference between R2 and Q2 (R2 − Q2) was calculated to reduce the possibility of overfitting in the supervised model [35]. If R2 − Q2 > 0.3, the model would be considered over-fitted and therefore invalidated.

The significance of the model was also evaluated using a permutation test [36]. The same procedure was repeated in this study 999 times (the maximum threshold in the SIMCA-P software version 14.1), and the parameters were compared to the original data parameters. The model was considered valid if the Q2 regression line crossed the zero line or if no Q2 value from the permutated data set was more than the Q2 from the original data set.

The significance of the group separation was assessed by using the *p*-value provided from the CV-ANOVA [37,38]. SIMCA-P produced this test based on a cross-validated model.

### 4.7. Data Filtration

In this study, several steps were applied to exclude metabolites with unreliable data points. The first filtering step was the *p*-value provided from the Student’s *t*-test. Metabolites with *p*-values > 0.05 were excluded from the list. The remaining metabolites were filtered using jack-knifing uncertainties. This filter evaluates the precision of each metabolite by estimating the prediction error rate after cross-validation. It can be provided by calculating the 95% confidence intervals (95% CI) from the supervised model [21]. Metabolites which registered zero within the 95% CI were excluded from the list.

The significant metabolites were transferred to Metaboanalyst (http://www.metaboanalyst.ca/) to compute the corrected *p*-value (*q*-value) and the area under the curve. The *p*-value was corrected using the Benjamini & Hochberg False Discovery Rate, and metabolites with *q*-values > 0.05 were excluded [39]. Area under the curves were tested for each of the significant metabolites, and the metabolites with areas < 0.7 were considered poor classifiers and excluded from the model [37]. A rough classification for areas under the curve is as follows: 0.9–1.0 = excellent classifier; 0.8–0.9 = good classifier; 0.7–0.8 = fair classifier; 0.6–0.7 = poor classifier; and 0.5–0.6 = failed classifier.

### 4.8. Ranking, Grouping and Confirmation of Significant Metabolites

The significant metabolites that passed the filtration steps were ranked by variable importance in the projection (VIP) values. VIP measures the contribution of each significant variable in the observed metabolomic change in a given model compared to that of the rest of the variables [35]. Metabolites with a VIP total > 1 were considered to have high contribution levels to the model [40]. In addition, confidence intervals on the VIP column plot should be positive [41]. The VIP values were divided into VIP predicted (VIPpred) and VIP orthogonal (VIPortho), where VIPpred represents the contribution of a metabolite to the difference between groups compared to the other metabolites, and VIPortho represents the contribution of a metabolite to the difference within groups compared to the other metabolites. The ratio of VIPpred/VIPortho was used in addition to the total VIP to evaluate metabolite contributions. Where VIPortho is > VIPpred a metabolite is not relevant as a biomarker.

The overall workflow of the study is summarised in Supplementary Figure S2. The main work flow was divided into five main steps, starting with sample analysis and data generation (blue), followed by data pre-processing and modelling (orange), model validation (yellow), metabolite filtering (purple) and finally ranking, grouping and conformation of the significant metabolites (green). Q-Q tests were conducted in Excel.

## 5. Conclusions

Several metabolomic differences were found in the faecal metabolome of paediatric patients with CD compared to the HC group. Thus, multivariate statistical methods were used to refine the marker list. An OPLS-DA model was able to separate all the CD groups throughout treatment and post-treatment from the HC group. However, it was not possible to obtain a valid model separating the CD groups throughout the different phases of treatment apart from between PA and PC. The eight markers which separated the CD groups from the HC groups were all normally distributed according to Q-Q tests. Large elevations in omega 6 fatty acids were observed in the CD patients in comparison with the HC group, conforming to previous work that highlighted these compounds as being pro-inflammatory in the gut. The results of this study indicate that major metabolic differences remained between the HC group the CD group even after apparently successful treatment; these metabolic differences could be clearly separated using multivariate statistical methods. The BMI values for the control group were not recorded and could impact on the results although this would seem more likely to occur for the metabolome of plasma rather than the fecal metabolome which is much more related to the activity of the microbiome. In future work with a larger set of samples, a linear model relying on the longitudinal nature of the samples will be applied in order to determine whether there are combinations of markers which are more indicative of the success of treatment.

## Figures and Tables

**Table 1:** An overview of all the orthogonal partial least square-discriminant analysis (OPLS-DA) parameters and their validity. The *p* CV-ANOVA column denotes the *p* value associated with the cross-validation analysis of variance (CV-ANOVA).

Model	R2X (Cum)	R2	Q2	Permutation (999 times)	R2 − Q2	Valid	*p* CV-ANOVA	Significance
PA vs. HC	0.63	0.95	0.71	yes	0.24	yes	1.83 × 10^−3^	yes
PA vs. PB	0.60	0.88	0.51	yes	0.37	no	1.37 × 10^−1^	no
PA vs. PC	0.65	0.88	0.66	yes	0.22	yes	1.00 × 10^−2^	yes
PA vs. PD	0.67	0.89	0.43	yes	0.46	no	2.42 × 10^−1^	no
PA vs. PE	0.47	0.67	0.33	yes	0.34	no	1.56 × 10^−1^	no
HC vs. PB	0.68	0.99	0.91	yes	0.08	yes	2.03 × 10^−6^	yes
HC vs. PC	0.67	0.99	0.91	yes	0.08	yes	4.81 × 10^−7^	yes
HC vs. PD	0.72	0.99	0.86	yes	0.13	yes	6.69 × 10^−4^	yes
HC vs. PE	0.54	0.99	0.72	yes	0.27	yes	1.19 × 10^−2^	yes
PB vs. PC	0.68	0.97	0.08	yes	0.89	no	9.97 × 10^−1^	no
PB vs. PD	0.61	0.76	0.12	yes	0.64	no	7.16 × 10^−1^	no
PB vs. PE	0.63	0.99	0.93	yes	0.06	yes	3.31 × 10^−7^	yes
PC vs. PD	0.58	0.68	0.24	yes	0.44	no	2.98 × 10-1	no
PC vs. PE	0.60	0.98	0.89	yes	0.09	yes	1.90 × 10^−7^	yes
PD vs. PE	0.57	0.84	0.69	yes	0.15	yes	3.43 × 10^−4^	yes

* (HC) Healthy control children, (PA) CD children pre-EEN treatment, (PB) CD children 15 days post-EEN treatment, (PC) CD children 30 days post-EEN treatment, (PD) CD children 60 days post-EEN treatment, (PE) CD children back to a free diet, (R2X (cum)) the cumulated amount of variation in matrix X, (R2) the goodness of fit, (Q2) the goodness of prediction.

**Table 2:** The relative abundance of long chain fatty acids in the faecal extracts based on analysis of a ZICp HILIC column.

Mass	RT	Putative Metabolite	*p*-Value HCPA	PA/HC	*p*-Value HCPB	PB/HC	*p*-Value HCPC	PC/HC	*p*-Value HCPD	D/HC	*p*-Value HCPE	E/HC
254.2246	3.6	Hexadecenoic acid	0.005	2.334	0.004	3.028	0.001	3.219	0.027	2.287	0.035	1.623
256.2401	3.6	Hexadecanoic acid	0.839	1.038	0.860	1.038	0.957	1.011	0.522	0.866	0.249	1.212
258.1829	3.6	Tetradecanedioic acid	0.016	0.362	0.010	0.332	0.007	0.300	0.068	0.507	0.637	1.200
258.2198	3.5	Hydroxypentadecanoic acid	0.984	1.005	0.713	1.143	0.401	1.379	0.266	1.448	0.716	1.095
260.1988	3.3	Dihydroxytetradecanoic acid	0.891	0.935	0.051	0.240	0.037	0.180	0.034	0.161	0.241	1.512
266.1882	3.4	Hydroxyhexadecatrienoic acid	0.537	0.839	0.007	0.391	0.015	0.459	0.029	0.506	0.192	1.387
268.2036	3.1	Hydroxyhexadecadienoic acid	0.016	0.376	0.001	0.118	0.001	0.113	0.002	0.176	0.552	0.852
270.2195	3.5	Hydroxyhexadecenoic acid	0.940	1.024	0.174	0.702	0.846	1.081	0.902	0.963	0.095	1.472
272.2351	3.5	Hydroxyhexadecanoic acid	0.905	0.947	0.228	0.578	0.389	0.690	0.538	0.785	0.449	1.287
278.2245	3.5	Octadecatrienoic acid	0.004	0.115	0.005	0.143	0.004	0.135	0.004	0.128	0.014	0.293
280.2401	3.6	Octadecadienoic acid	0.212	0.513	0.220	0.517	0.211	0.508	0.098	0.342	0.195	0.500
282.2559	3.6	Octadecenoic acid	0.826	1.093	0.374	0.666	0.839	1.102	0.407	0.688	0.065	1.871
284.2713	3.5	Octadecanoic acid	0.399	0.756	0.022	0.400	0.020	0.394	0.018	0.374	0.928	0.976
288.23	3.2	Dihydroxyhexadecanoic acid	0.810	0.893	0.047	0.252	0.048	0.256	0.052	0.269	0.296	1.436
296.2349	3.5	Hydroxyoctadecadienenoic acid	0.004	0.285	0.004	0.271	0.004	0.269	0.004	0.279	0.235	0.700
298.2506	3.6	Hydroxyoctadecenoic acid	0.797	1.105	0.509	0.784	0.921	1.038	0.950	1.023	0.019	2.064
304.2401	3.5	Eicosatetraenoic acid	0.088	18.052	0.100	4.904	0.049	2.915	0.228	1.968	0.008	3.448
306.2558	3.5	Eicosatrienoic acid	0.002	16.182	0.008	13.854	0.014	9.671	0.049	8.821	0.003	8.751
308.2715	3.5	Eicosadienoic acid	0.028	8.716	0.041	6.338	0.017	6.119	0.008	5.658	0.000	3.709
310.2145	3.5	Dihydroxyoctadecatrienoic acid	0.042	0.593	0.691	0.890	0.368	0.783	0.017	0.496	0.676	1.147
310.2871	3.5	Eicosenoic acid	0.045	1.793	0.308	1.315	0.422	1.181	0.772	1.076	0.020	1.588
312.2301	3.6	Dihydroxyoctadecadienoic acid	0.871	1.053	0.868	1.053	0.860	0.947	0.191	0.647	0.350	1.244
312.2663	3.5	Hydroxynonadecenoic acid	0.232	1.695	0.013	2.360	0.018	2.560	0.045	2.148	0.733	1.149
312.3028	3.5	Eicosanoic acid	0.851	1.071	0.203	1.595	0.082	2.166	0.304	1.614	0.766	1.085
330.2405	3.7	Trihydroxyoctadecenoic acid	0.175	0.508	0.373	1.590	0.646	1.220	0.878	0.938	0.583	1.261
332.2716	3.5	Docosatetraenoic acid	0.006	20.326	0.004	9.694	0.003	6.946	0.006	6.532	<0.001	8.116
334.2144	3.7	Dihydroxyeicosapentaenoic acid	0.964	1.024	0.938	1.043	0.661	0.775	0.605	0.743	0.233	0.414
334.2871	3.5	Docosatrienoic acid	0.127	1.701	0.491	1.457	0.856	1.098	0.405	1.491	0.201	1.664
336.3029	3.5	Docosadienoic acid	0.738	1.226	0.444	0.705	0.037	0.341	0.116	0.488	0.880	0.939
338.3186	3.5	Docosenoic acid	0.119	1.665	0.768	0.935	0.192	0.759	0.185	0.728	0.039	1.459
340.334	3.4	Docosanoic acid	0.610	1.264	0.017	0.320	0.019	0.342	0.026	0.356	0.262	1.570
342.2769	3.4	Eicosanedioic acid	0.021	0.311	0.061	0.454	0.069	0.468	0.088	0.504	0.331	0.718
346.2353	3.9	Tetrahydroxyoctadecenoic acid	0.046	0.447	0.301	0.691	0.168	0.618	0.244	0.670	0.978	0.992
352.3341	3.4	Tricosenoic acid	0.164	1.402	0.006	2.492	0.005	2.596	0.127	1.788	0.088	1.392
354.2408	3.7	Trihydroxyeicosatetraenoic acid	0.799	0.938	0.254	0.697	0.078	0.598	0.239	0.730	0.097	1.433
354.3134	3.4	Hydroxydocosenoic acid	0.209	0.563	0.022	0.361	0.036	0.419	0.088	0.529	0.602	1.160
354.3498	3.4	Tricosanoic acid	0.999	1.000	0.011	0.351	0.019	0.427	0.019	0.408	0.511	1.225
356.329	3.4	Hydroxydocosanoic acid	0.494	0.713	0.011	0.239	0.013	0.270	0.021	0.325	0.461	0.763
364.3342	3.4	Tetracosadienoic acid	0.037	4.794	0.214	2.076	0.651	1.220	0.312	2.154	0.048	3.661
370.2358	3.8	Tetrahydroxyeicosatrienoic acid	0.456	0.840	0.044	0.532	0.012	0.464	0.039	0.536	0.075	1.544
372.2509	3.8	Tetrahydroxyeicosadienenoic acid	0.086	0.574	0.030	0.537	0.004	0.402	0.016	0.497	0.080	1.459
382.2719	3.6	Dihydroxydocosatrienoic acid	0.039	0.268	0.101	0.379	0.041	0.278	0.140	0.459	0.131	0.485
382.3447	3.3	Hydroxy tetracosanoic acid	0.556	0.702	0.090	0.282	0.084	0.270	0.121	0.349	0.835	1.103

(HC) Healthy control children, (PA) CD children pre-EEN treatment, (PB) CD children 15 days post-EEN treatment, (PC) CD children 30 days post-EEN treatment, (PD) CD children 60 days post-EEN treatment, (PE) CD children back to a free diet.

**Table 3:** List of metabolites that were significantly different in the pre-EEN treatment group (PA) compared to the healthy controls (HC), based on an OPLS-DA model. All marker compounds were normally distributed according to a Q-Q test.

Putative Metabolite	Pathway	(PA/HC)	*p*-Value	*q*-Value	AUC	VIP Total	VIP (Pred./Ortho.)
Ornithine isomer	unknown	0.15	7.82 × 10^−3^	2.67 × 10^−2^	0.84	1.85	4.28
C20 sphingenine	Sphingoid bases	6.54	2.03 × 10^−2^	3.92 × 10^−2^	0.75	1.81	2.82
Tyrosine	Tyrosine metabolism	0.37	2.64 × 10^−2^	4.98 × 10^−2^	0.83	1.63	1.84
SM (d18:1/24:1)	Ceramide phosphocholines (sphingomyelins)	14.52	3.28 × 10^−3^	2.67 × 10^−2^	0.87	1.26	1.08
Eicosatrienoic acid	Biosynthesis of unsaturated fatty acids	16.18	3.48 × 10^−4^	4.67 × 10^−3^	0.88	1.07	1.53
Docosatetraenoic acid	Biosynthesis of unsaturated fatty acids	20.32	9.11 × 10^−4^	6.15 × 10^−3^	0.92	1.02	1.01
Arachidonic acid	Fatty Acids and Conjugates	18.05	4.79 × 10^−3^	1.94 × 10^−2^	0.88	0.99	2.91
Octadecenoylsphingenine	Ceramides	11.88	5.31 × 10^−5^	1.08 × 10^−3^	0.94	0.89	1.21

**Table 4:** Details of characterization of the eight marker compounds shown in Table 1 obtained in positive (+) or negative (−) ion mode. MS^n^ fragments obtained at 30 V collision energy for three of the marker compounds shown in Table 3 obtained by using an Orbitrap Fusion mass spectrometer at 50000 resolution in MS^2^ mode and low resolution in MS^3^ mode. Chromatography carried out on ZICpHILIC or and ACE C4 column (C4).

*m/z*	Rt min	Elemental Composition	Putative ID	Deviation ppm	MS^2^/MS^3^	Comments
133.0971 (+)	87	C_5_H_13_O_2_N_2_	Ornithine isomer	−0.332	MS^2^ 115.085 (C_5_H_11_ON_2_), 98.060 (C_5_H_8_ON), 69.033 (C_4_H_5_NO)	Nearest alternative composition C_3_H_11_N_5_O (+9.8 ppm) Despite the MS^2^ fragments making sense (Figure S4), it is difficult to propose a definitive structure.
328.3211 (+)	3.4	C_20_H_42_O_2_N	C20 sphinganine	+0499	MS^2^ 311.2943 (C_20_H_39_O_2_),310.30951 (C_20_H_42_ON) 228.1957(C_13_H_26_O_2_N) 188.1644 (C_10_H_22_O_2_N)	Proposed fragmentation scheme shown in Figure S5. (Spectrum Figure S6).
813.6851 (+)	3.3	C_47_H_94_N_2_PO_6_	Ceramide d18:1 24:1	−1.145	MS^2^ 795.61, 553.53 MS^3^ (7956) 777.3, 614.6, 495.22, 264.1	This marker remains unidentified since it is not possible to relate the fragments to the proposed structure. Nearest alternative composition. Nearest match C51H91NO6 (1.5 ppm). MS^2^ and MS^3^ spectra Figures S7 and S8.
182.0810 (+)	13.5	C_9_H_12_NO_3_	Tyrosine	−0.810	-	Matches retention time of standard. Nearest alternative composition C_7_H_10_NO_2_ (+7.9 ppm)
305.2484 (−)	19.5 C4	C_20_H_33_O_2_	Eicosatrienoic acid	−0.438	-	Matches retention time of standard. Nearest alternative composition C_18_H_31_ON_3_ (+3.9 ppm)
329.2484 (−)	191 C4	C_22_H_33_O_2_	Docosapentaenoic acid	−0.406	-	No standard available but logically the retention time falls close to eicosatrienoic acid because number of hydrogens is the same.
303.2329 (−)	18.5 C4	C_20_H_31_O_2_	Arachidonic acid	−0.045	-	Matches retention time of standard. Nearest alternative composition C_18_H_29_ON_3_ (+4.3 ppm)
564.5361 (+)	3.1	C_36_H_70_NO_3_	Octadecenoylsphinganine	+19.8	MS^2^ 546.5239(C_36_H_68_NO_2_) 528.5128 (C_36_H_66_NO) 282.2782 (C_18_H_38_NO) 264.2680 (C_18_H_36_N)	Proposed fragmentation scheme shown in Figure S9. MS^2^ spectrum Figure S10.

**Table 5:** Concentration of fatty acids in each sample (µg/g of dry faeces).

**Healthy Controls**		
**Sample**	**Arachidonic Acid**	**Cis-8, 11, 14-Eicosatrienoic acid**
HC01	47.6	112.4
HC02	13.6	10
HC03	25.6	19.6
HC04	7.6	3.6
HC05	15.2	2
HC06	63.6	210.4
HC07	86.8	136.4
HC08	144.8	127.6
HC09	12.8	5.6
HC10	13.2	16.4
HC11	34.8	60
Mean	42.4	64
SD	42.4	71.6
SEM	12.8	21.6
**Crohn’s disease**		
**Sample**	**Arachidonic Acid**	**Cis-8, 11, 14-Eicosatrienoic acid**
PA01	4406	3854.4
PA02	4365.2	1671.6
PA03	432.4	492.4
PA04	1644.8	9462.4
PA05	92	514.4
PA06	3510	3600.4
PA07	98	196
PA08	44.8	904.8
PA09	27.6	181.6
PA10	14.0	50.0
PA11	4262.8	1029.2
Mean	1718	1996
SD	1985.2	2806.8
SEM	598.4	846.4
* *p*-value	0.019	0.046

* Based on log2 values.

**Table 6:** Samples numbers and groups of paediatric Crohn’s disease before, during, and after EEN and healthy controls.

Group ID	Description	*n*
PA	CD children pre-EEN treatment	11
PB	CD children 15 days of EEN treatment	10
PC	CD children 30 days of EEN treatment	11
PD	CD children 60 days of EEN treatment	11
PE	CD children back to normal diet	11
HC	Healthy children control	11

**Table 7:** Subject data for healthy controls and patients. na = not recorded, nr = not relevant. PCDAI = Paediatric Disease Activity Index.

Subjects	Sex	Age	BMI at Enrolment (kg/m^2^)	Weight (kg) at Enrolment (kg/m^2^)	BMI Z Score at Enrolment (kg/m^2^)	BMI (kg/m^2^) at 4 Weeks	Weight (kg) at 4 Weeks	BMI Z Score Increase 4 Weeks	BMI (kg/m^2^) at 8 Weeks	Weight (kg) at 8 Weeks	BMI Z Score Increase 8 Weeks	Treatment Naïve	Previously Treated	PCDAI at Start	PCDAI at End
CD Patients	4 F 7 M	11.5 ± 2.4	13.8 ± 1.4	28.9 ± 6.0	−1.61 ± 0.27	15.7 ± 1.3	30.8 ± 6.3	1.6 ± 0.38	16.2 ± 1.5	33.3 ± 5.2	1.7 ± 0.35	7	4	11 > 10	7 < 10
Healthy controls	4 F 7 M	10.2 ± 2.3	na	na	na	nr	nr	nr	nr	nr	nr	nr	nr	nr	nr

## Supplementary Materials

The following are available online at http://www.mdpi.com/2218-1989/8/4/82/s1, Figure S1: OPLSDA plots and model validation, Figure S2: Flow chart for sample and data analysis, Figure S3: QQ Plots for the marker compounds and some compounds reported in Table S1 as significant but not normally distributed. Table S1: Small polar marker compounds. Table S2: Sphingosine metabolism. Table S3 Calprotectin values. Figure S4: MS2 fragments of ornithine isomer. Figure S6: Proposed fragmentation of C20 sphinganine. Figure S7: MS2 spectrum of C20 sphinganine. Figure S8: MS2 spectrum of Ceramide d18:1 24:1. Figure S9: MS3 spectrum of Ceramide. d18:1 24:1 (795.5 ion). Figure S10: Proposed fragmentation of octadecenoylsphingenine. Figure S11: MS2 spectrum of octadecylsphinganine. Table S4: Subject data for healthy controls and patients. na= not recorded.
