# Supplementary material for: Untargeted Metabolomics of Extracts from Faecal Samples Demonstrates Distinct Differences between Paediatric Crohn’s Disease Patients and Healthy Controls but No Significant Changes Resulting from Exclusive Enteral Nutrition Treatment
Source: Metabolites. 2018 Nov 22;8(4):82. doi: 10.3390/metabo8040082 (PMC6315767; doi:10.3390/metabo8040082)
Supplement: Supplementary file 1 [file metabolites-08-00082-s001.pdf]

metabolites

MDPI

Untargeted metabolomics of extracts from faecal samples demonstrates distinct differences between paediatric Crohn's disease patients and healthy controls but no significant changes resulting from exclusive enteral nutrition treatment.

Adel Alghamdi [1], Konstantinos Gerasimidis* [2]†, Gavin Blackburn[3], Didem Akinci[3], Christine Edwards[3], Richard K. Russell[4] and David G. Watson[1]*

[1] Strathclyde Institute of Pharmacy and Biomedical Sciences, University of Strathclyde, 161, Cathedral Street, Glasgow, G4 0RE.
[2] School of Medicine, College of MVLS, University of Glasgow, Room 3.09, Level 3, New Lister Building, Glasgow Royal Infirmary, 10-16 Alexandra Parade, G31 2ER;
[3] Glasgow Polyomics, Glasgow Polyomics Metabolomics Facility ManagerTranslational Cancer Research CentreUniversity of Glasgow Garscube Campus,Switchback Road,Glasgow G61 1QH
[4] Department of Paediatric Gastroenterology, Hepatology and Nutrition, Royal Hospital for Children, 1345 Govan Road, Glasgow, G51 4TF;
* Correspondence: d.g.watson@strath.ac.uk; Tel.: +44-141-548-2651; konstantinos.gerasimidis@glasgow.ac.uk
† Shared first authorship

Supplementary Material

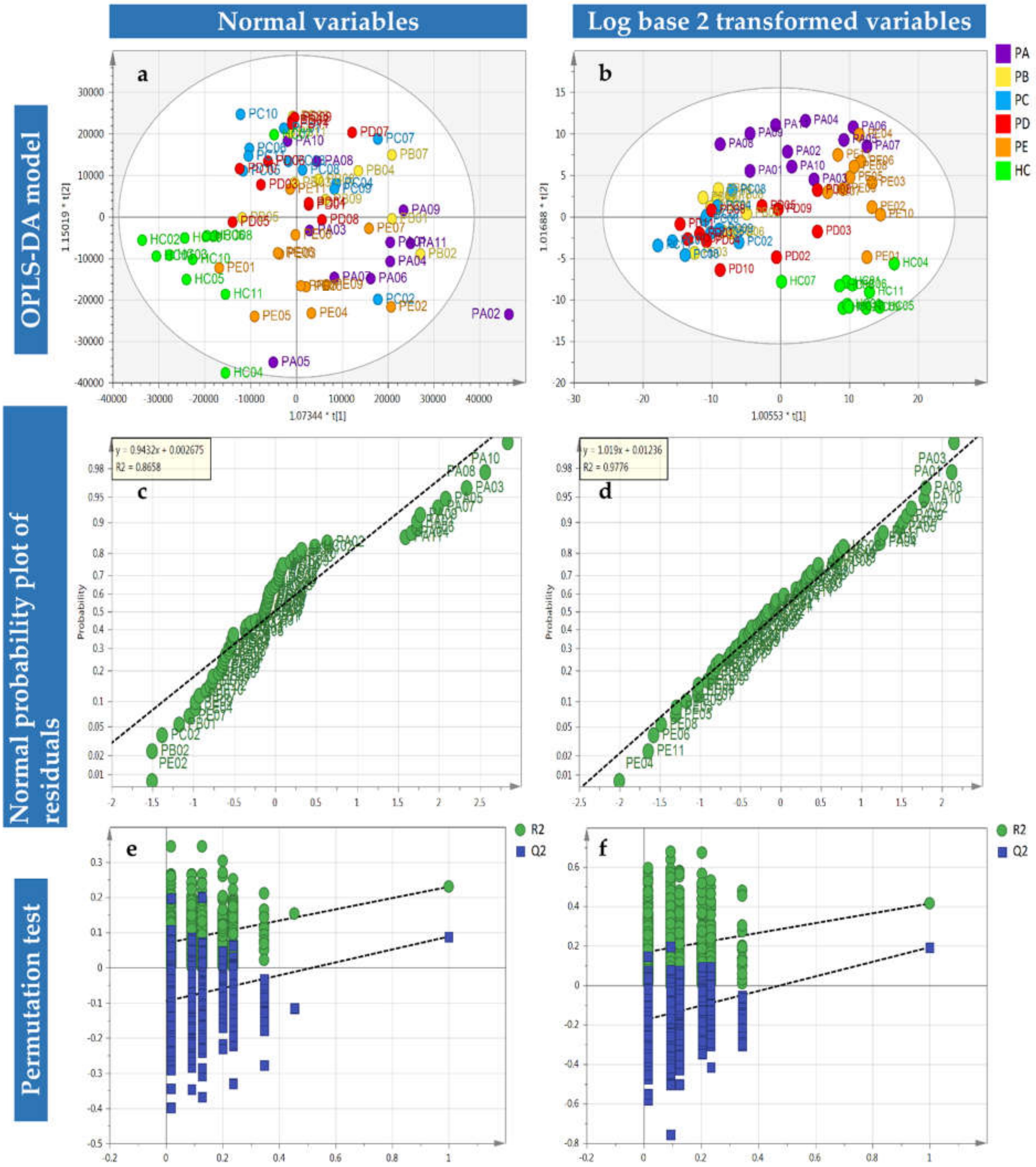

Figure S1. The effect of log base 2 transformation on the model separation and validation: (**a**) the 2D Orthogonal Partial Least Square-Discriminant Analysis (OPLS-DA) model score plot for the normal variables; and (**b**) a plot of the log2 transformed variables. All the variables were Pareto scaled, and the OPLS-DA model was based on 376 putative metabolites. Sample groups: (HC) healthy control children, (PA) CD children pre-EEN treatment, (PB) CD children 15 days during EEN treatment, (PC) CD children 30 days during EEN treatment, (PD) CD children 60 days during EEN treatment and (PE) CD children back to their habitual free diet. The data was further analysed with: (**c**) a normal probability plot of the residuals for the normal variables; and (**d**) a plot of the log2 transformed variables. Plot **c** and **d** display the residuals standardized on a double log scale along the y-axis versus the standard deviation on the x-axis. Outlier variables are displayed outside -4 to 4 standard deviation intervals. The regression line assessed the normality of the residuals. A permutation test (999 times) was run for the OPLS-DA model based on all the samples: (**e**) the plot of the normal variables; and (**f**) the plot of the log2

transformed variables. The OPLS-DA model is considered valid if the regression line of the goodness of prediction (Q2) points intersects the vertical axis (on the left) at or below zero.

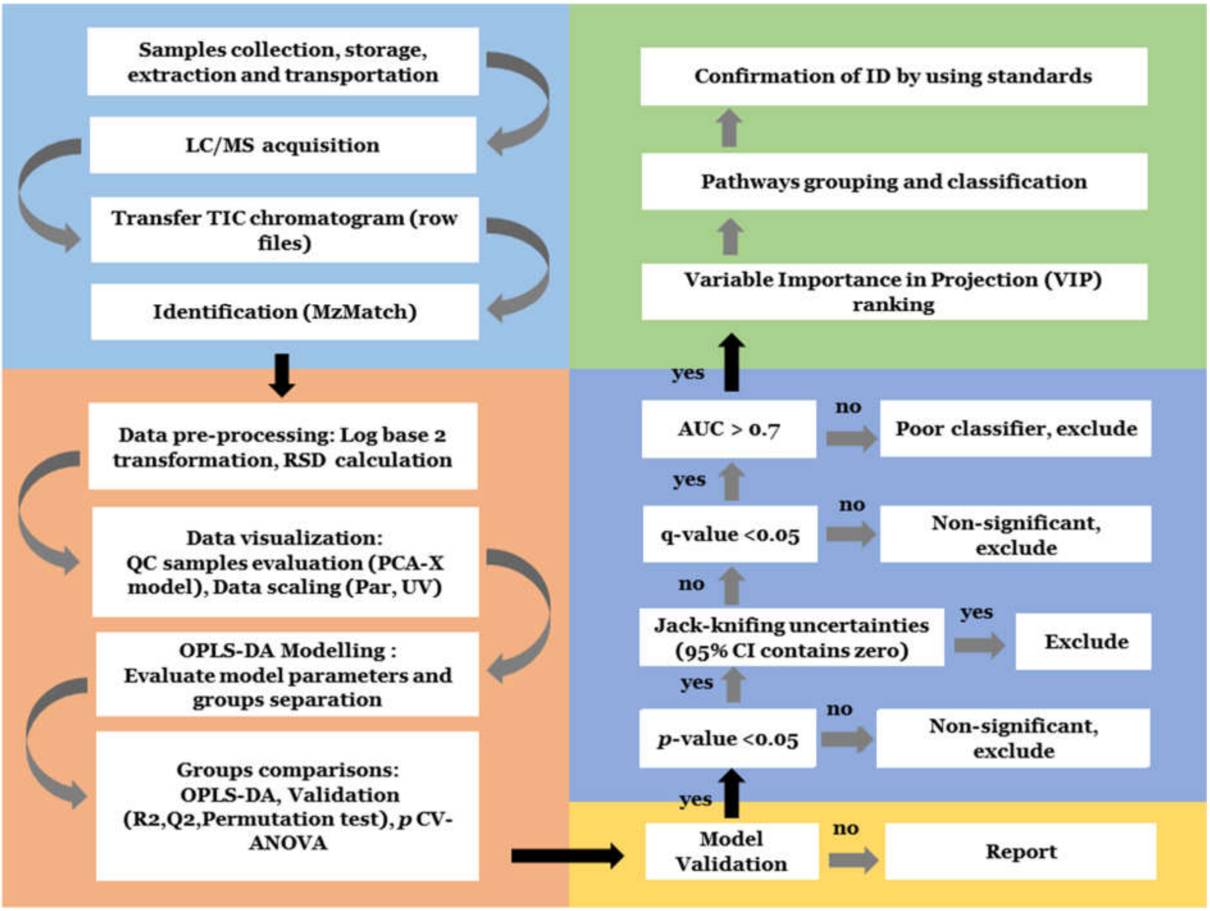

Figure S2 Flow chart for sample and data analysis

Figure S3 QQ Plots for the marker compounds and some compounds reported in table S1 as significant but not normally distributed.

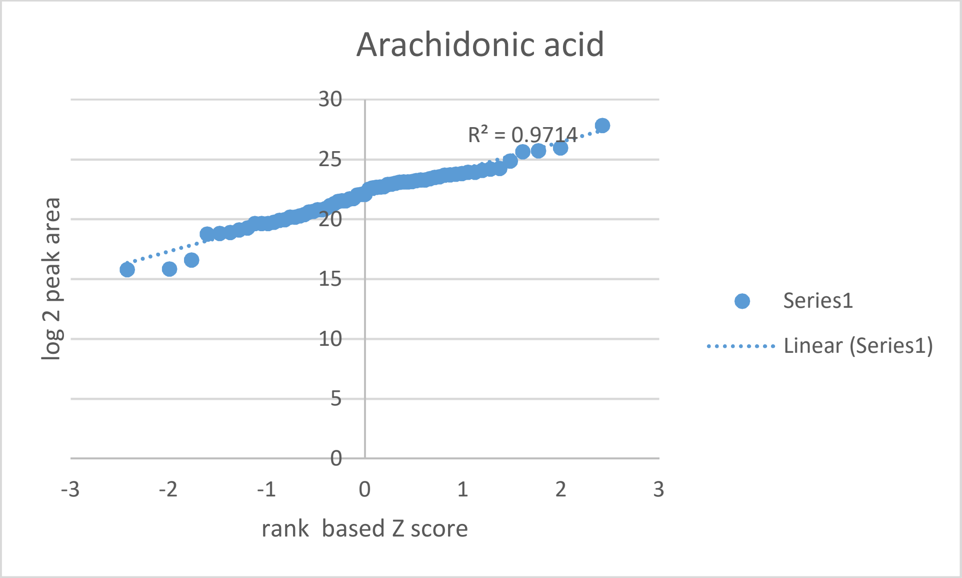

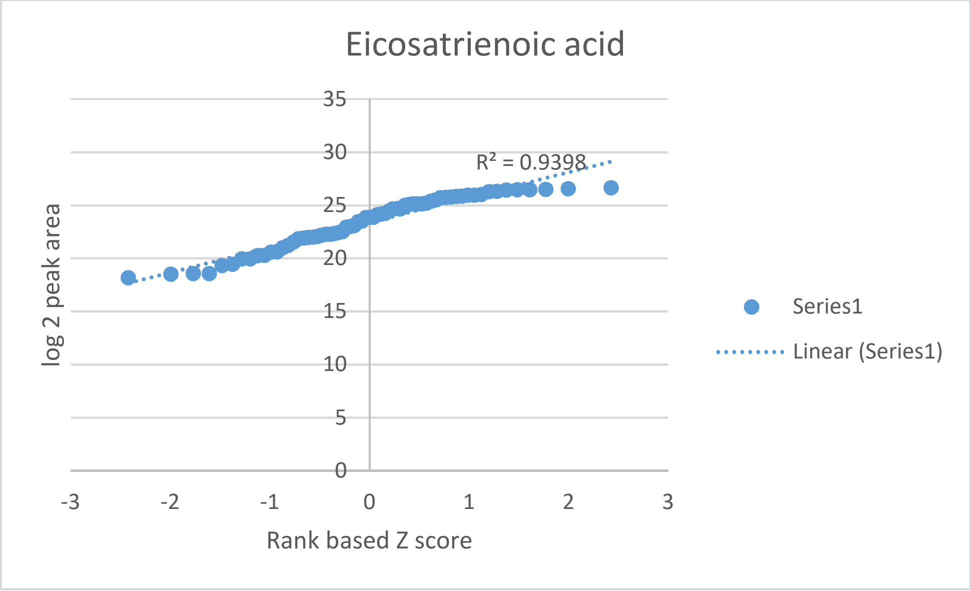

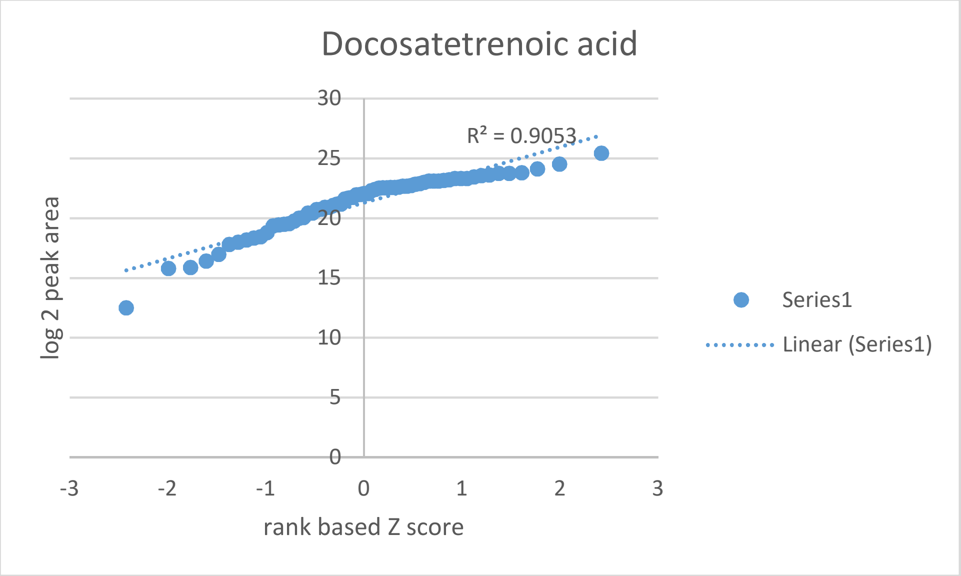
Docosatetrenoic acid
log 2 peak area
rank based Z score
R² = 0.9053
Series1
Linear (Series1)

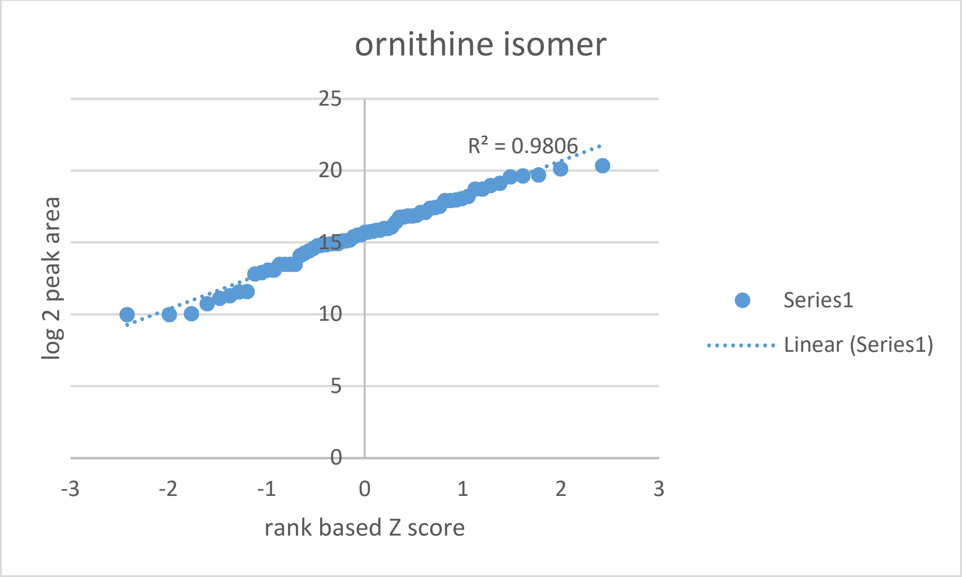
ornithine isomer
log 2 peak area
rank based Z score
R² = 0.9806
Series1
Linear (Series1)

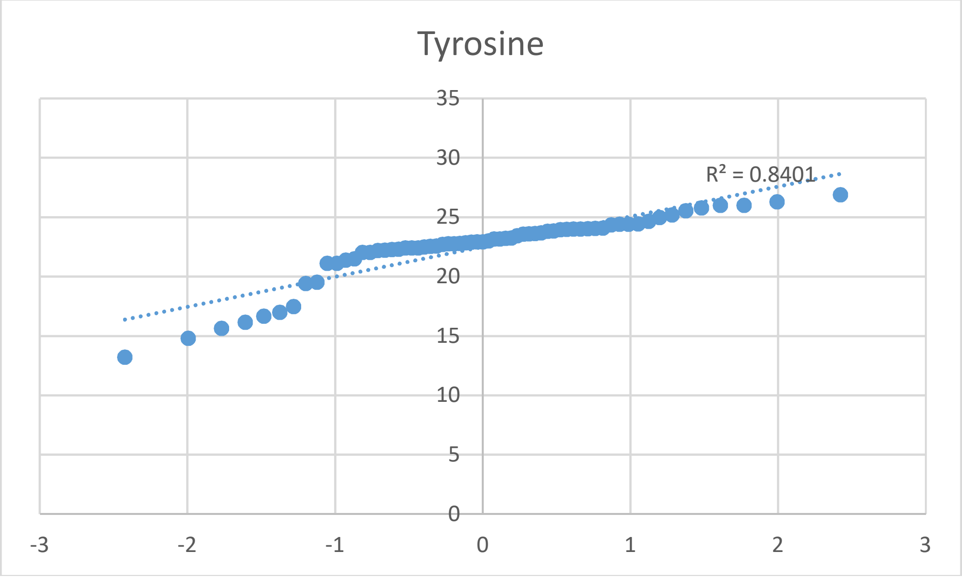
Tyrosine
R² = 0.8401

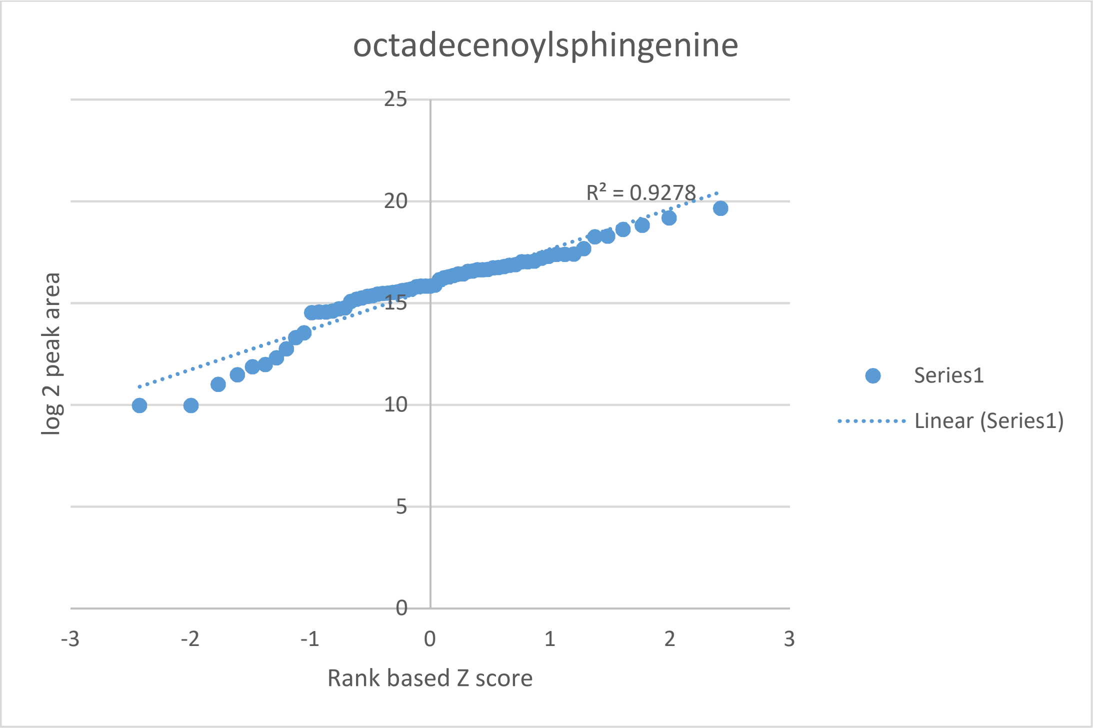
octadecenoylsphingenine
log 2 peak area
Rank based Z score
R² = 0.9278
25
20
15
10
5
0
-3
-2
-1
0
1
2
3
Series1
Linear (Series1)

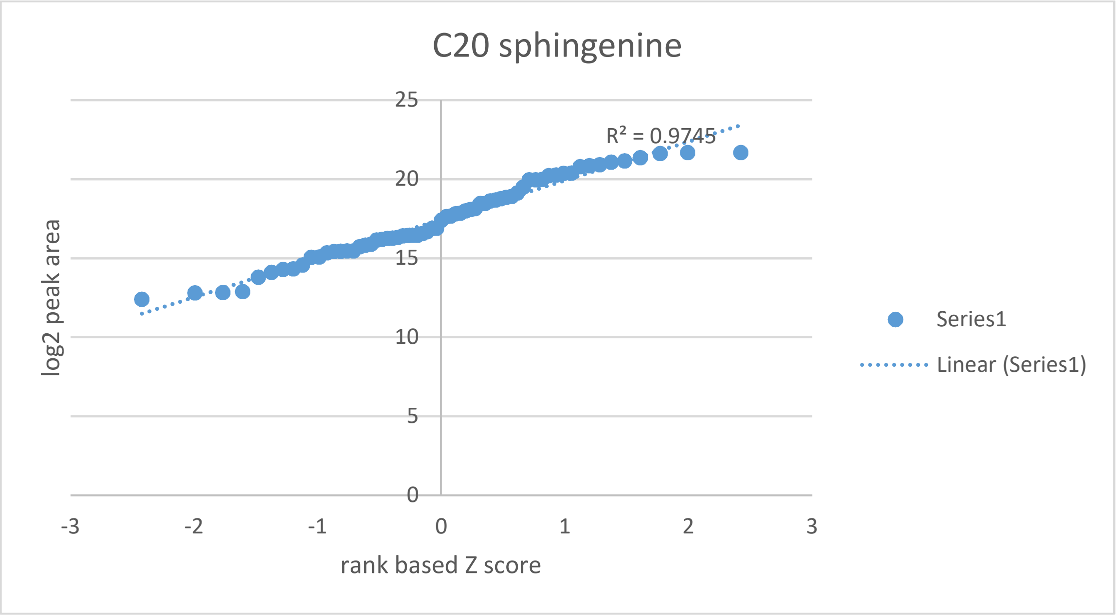
C20 sphingenine
log2 peak area
rank based Z score
R² = 0.9745
25
20
15
10
5
0
-3
-2
-1
0
1
2
3
Series1
Linear (Series1)

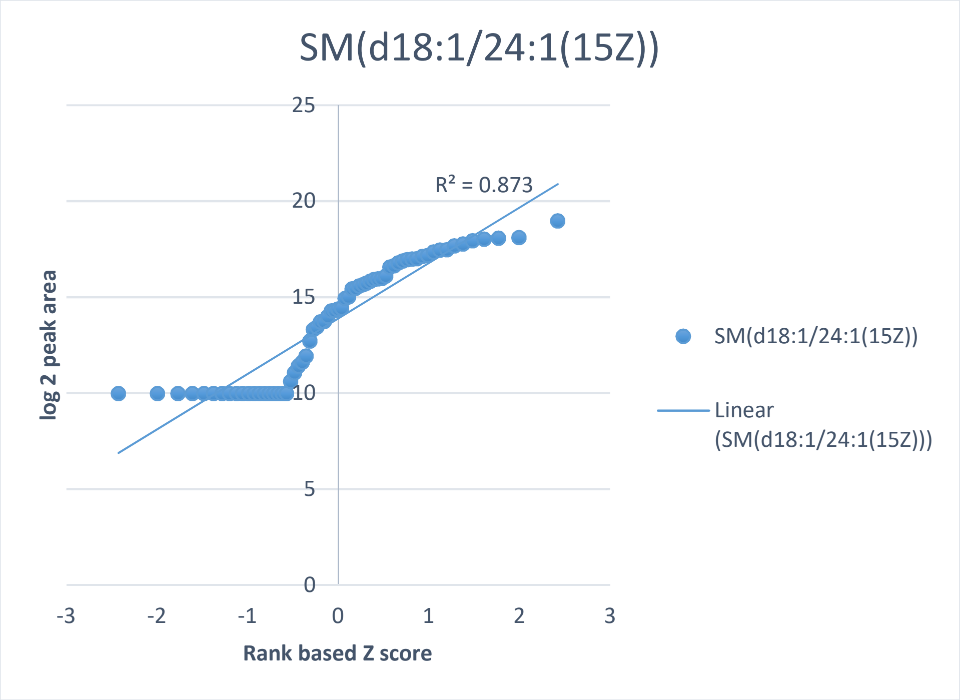
SM(d18:1/24:1(15Z))
R² = 0.873
log 2 peak area
Rank based Z score
SM(d18:1/24:1(15Z))
Linear (SM(d18:1/24:1(15Z)))
25
20
15
10
5
0
-3
-2
-1
0
1
2
3

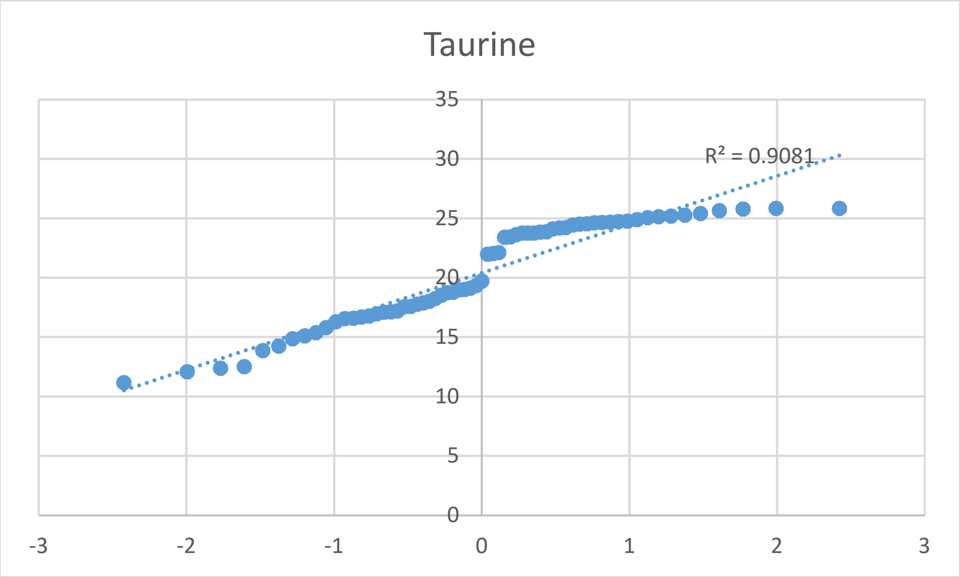
Taurine
R² = 0.9081
35
30
25
20
15
10
5
0
-3
-2
-1
0
1
2
3

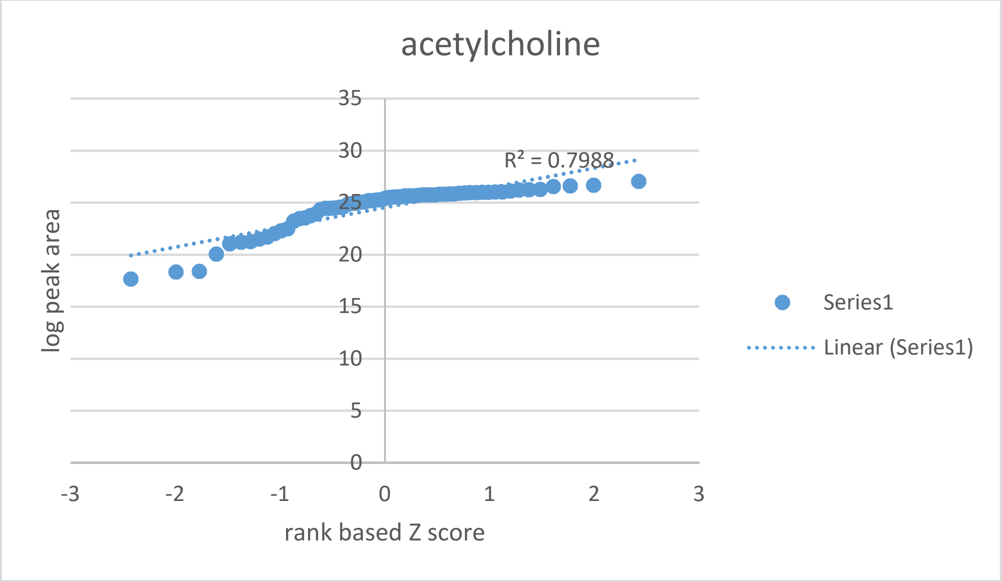
acetylcholine
log peak area
35
30
25
20
15
10
5
0
-3
-2
-1
0
1
2
3
rank based Z score
R² = 0.7988
Series1
Linear (Series1)

Appendix Table S1 Small polar marker compounds. * Corresponds to the retention time of a standard.

Mass	RT	Putative metabolite	P value HC/PA	PA/HC	P value HC/PB	PB/PC	P value HC/PC	PC/HC	p value HC/PD	PD/HC	p value HE/PC	PE/HC
75.03215	16.2	*Glycine	0.111	3.885	0.399	1.804	0.755	1.234	0.565	1.171	0.415	1.268
88.01596	8.3	*Pyruvate	0.694	0.727	0.319	0.338	0.211	0.169	0.217	0.183	0.951	0.954
88.05236	6.6	Butanoic acid	0.356	1.880	0.853	0.901	0.161	0.422	0.151	0.415	0.406	1.694
89.04762	15.3	*Alanine	0.089	3.464	0.212	2.170	0.769	1.176	0.640	0.884	0.091	1.646
89.04774	15.9	*beta-Alanine	0.028	5.995	0.140	4.787	0.273	2.699	0.362	2.325	0.097	5.351
92.04734	10.7	Glycerol	0.112	0.191	0.096	0.151	0.070	0.066	0.078	0.093	0.107	0.182
97.96741	8.8	Sulfate	0.459	1.412	0.436	1.343	0.442	1.389	0.750	0.900	0.053	2.214
103.0633	14.3	N,N-Dimethylglycine	0.029	4.109	0.096	4.191	0.102	2.577	0.057	2.938	0.015	4.437
103.0633	12.8	*3-Amino-isobutanoate	0.006	3.129	0.246	1.390	0.457	1.218	0.615	1.163	0.038	1.549
103.0634	16.1	*3-Amino-isobutanoate	0.527	0.739	0.463	0.629	0.046	0.185	0.044	0.180	0.158	2.253
104.011	8.0	Hydroxypyruvate	0.324	12.871	0.030	11.386	0.035	7.553	0.044	6.936	0.146	6.110
104.0474	7.6	4-Hydroxybutanoic acid	0.291	15.200	0.069	4.222	0.326	1.723	0.610	0.788	0.038	2.717
105.0427	16.3	*Serine	0.187	7.201	0.199	2.962	0.285	0.731	0.944	0.980	0.173	1.618
109.0528	10.5	2-Aminophenol	0.950	1.027	0.001	0.312	0.001	0.298	0.976	1.009	0.009	2.053
109.0528	7.7	4-Hydroxyaniline	0.258	124.161	0.372	12.058	0.236	27.380	0.309	52.264	0.268	82.550
111.032	8.0	Pyrrole-2-carboxylate	0.244	0.438	0.149	0.304	0.126	0.256	0.792	0.829	0.520	1.311
111.032	9.8	Pyrrole-2-carboxylate	0.069	0.214	0.044	0.115	0.041	0.096	0.985	0.982	0.698	0.785
112.0273	8.2	Uracil	0.185	3.869	0.012	6.376	0.086	5.519	0.019	3.647	0.396	1.470
112.0273	8.8	Uracil	0.765	1.124	0.041	0.456	0.001	0.215	0.018	0.434	0.622	0.854
113.0477	10.8	1-Pyrroline-2-carboxylate	0.087	1.753	0.015	3.122	0.012	2.828	0.024	3.286	0.209	1.183
113.0478	16.4	(S)-1-Pyrroline-5-carboxylate	0.016	2.333	0.159	1.678	0.988	1.004	0.225	1.452	0.027	1.732
114.0318	15.0	2-Hydroxy-2,4-pentadienoate	0.174	2.606	0.856	0.904	0.111	0.375	0.208	0.509	0.154	1.720
114.043	9.4	5,6-Dihydrouracil	0.407	1.275	0.839	1.079	0.335	0.755	0.785	0.925	0.152	1.379
115.0269	8.1	Maleamate	0.800	0.930	0.625	0.873	0.350	0.741	0.401	0.779	0.082	2.041

115.0269	9.5	Maleamate	0.207	1.716	0.101	2.092	0.200	1.613	0.315	1.801	0.006	2.565
115.0269	10.7	Maleamate	0.142	13.497	0.108	3.270	0.091	2.270	0.322	3.582	0.024	3.793
115.0633	13.3	*Proline	0.134	2.731	0.231	2.617	0.491	1.457	0.773	1.115	0.521	1.202
115.0633	9.0	3-acetamidopropanal	0.133	3.748	0.829	0.908	0.799	0.878	0.604	0.788	0.024	3.095
115.0634	16.5	Proline	0.138	4.628	0.253	4.269	0.399	1.928	0.363	1.319	0.008	1.673
117.0426	8.0	L-2-Amino-3-oxobutanoic acid	0.114	3.607	0.045	2.314	0.479	1.191	0.081	1.679	0.049	2.198
117.0579	10.7	Indole	0.017	4.495	0.094	3.169	0.149	1.859	0.076	1.954	0.055	2.180
117.0579	6.2	Indole	0.330	1.478	0.877	0.942	0.603	1.231	0.835	1.074	0.143	1.806
117.0789	13.0	[FA amino(5:0)] 2S-amino-pentanoic acid	0.027	2.649	0.225	1.626	0.982	1.008	0.367	1.292	0.129	1.564
117.0789	16.3	5-Aminopentanoate	0.017	3.414	0.089	2.193	0.242	1.663	0.373	1.401	0.017	3.043
117.0789	12.4	* Valine	0.047	2.205	0.425	1.345	0.584	0.833	0.736	1.097	0.647	1.136
117.079	11.9	* Betaine	0.037	0.382	0.002	0.084	0.002	0.055	0.005	0.185	0.454	1.324
118.063	7.8	5-Hydroxypentanoate	0.966	1.029	0.282	2.119	0.896	0.902	0.574	0.663	0.438	0.573
118.063	5.2	formyl 3-hydroxy-butanoate	0.122	2.609	0.161	1.951	0.529	1.434	0.866	1.096	0.756	1.148
119.0583	14.9	*Threonine	0.165	8.056	0.177	3.783	0.585	1.413	0.407	1.346	0.066	1.736
122.0368	7.8	Benzoate	0.146	2.946	0.189	6.681	0.142	3.795	0.346	2.525	0.097	4.791
122.048	7.9	*Nicotinamide	0.057	5.263	0.165	5.236	0.264	2.131	0.331	2.433	0.110	1.731
123.032	7.8	*Nicotinate	0.826	0.858	0.547	1.515	0.396	0.444	0.520	0.576	0.772	1.203
123.0321	13.6	Nitrobenzene	0.823	1.085	0.215	3.501	0.116	2.650	0.296	1.875	0.153	2.265
125.0146	15.0	*Taurine	0.002	10.929	0.052	5.090	0.109	3.540	0.544	1.678	0.049	4.579
125.0589	10.6	5-Methylcytosine	0.207	7.537	0.023	4.033	0.123	3.947	0.046	4.868	0.009	4.949
126.0429	8.3	Thymine	0.034	8.118	0.110	2.911	0.571	1.296	0.362	1.578	0.030	3.857
126.0429	7.8	Thymine	0.274	1.780	0.284	0.644	0.010	0.276	0.021	0.349	0.613	0.828
127.0633	14.0	2,3,4,5-Tetrahydropyridine-2-carboxylate	0.010	2.675	0.008	2.518	0.091	1.722	0.041	2.215	0.013	2.433
128.0585	9.6	gamma-Amino-gamma-cyanobutanoate	0.039	2.431	0.188	3.074	0.258	3.088	0.180	2.639	0.007	2.125

128.0585	15.4	5,6-Dihydrothymine	0.255	18.902	0.299	4.904	0.150	3.277	0.324	1.670	0.427	1.469
129.0426	8.3	Oxoproline	0.758	1.158	0.800	1.205	0.967	1.028	0.325	0.755	0.226	0.695
129.0789	12.8	N4-Acetylaminobutanal	0.827	0.933	0.034	0.435	0.071	0.495	0.108	0.552	0.724	0.907
129.079	11.6	L-Pipecolate	0.492	0.750	0.005	0.142	0.004	0.130	0.039	0.344	0.341	1.416
129.0791	12.0	N4-Acetylaminobutanal	0.933	1.032	0.030	0.331	0.041	0.348	0.077	0.414	0.222	1.731
130.063	4.6	4-Methyl-2-oxopentanoate	0.338	4.148	0.212	2.370	0.692	0.827	0.606	1.245	0.746	1.188
131.0582	9.5	N-Acetyl-beta-alanine	0.147	3.701	0.156	1.622	0.646	1.150	0.735	1.109	0.099	1.568
131.0582	14.9	N-Acetyl-beta-alanine	0.525	1.348	0.179	0.494	0.018	0.392	0.000	0.175	0.177	2.154
131.0694	15.4	*Creatine	0.081	62.697	0.276	11.737	0.165	30.663	0.141	1.776	0.319	23.801
131.0945	11.8	*Leucine	0.056	3.832	0.146	2.322	0.479	1.413	0.109	1.726	0.094	1.686
131.0946	11.4	*Isoleucine	0.068	4.854	0.131	3.337	0.484	1.730	0.153	1.647	0.126	2.247
132.0423	8.1	2-Acetolactate	0.535	1.349	0.960	1.027	0.273	0.587	0.552	0.765	0.191	1.670
132.0533	11.9	N-Carbamoylsarcosine	0.149	9.585	0.215	6.575	0.626	1.555	0.975	1.012	0.035	1.937
132.0534	8.4	3-Ureidopropionate	0.330	6.095	0.397	1.646	0.341	0.688	0.763	1.132	0.490	1.219
132.0535	15.6	*Asparagine	0.253	33.837	0.303	8.977	0.328	15.753	0.268	2.507	0.231	2.931
132.0786	4.1	hydroxy-isocaproic acid	0.265	2.789	0.507	1.682	0.531	1.795	0.646	0.800	0.706	0.867
132.0898	8.7	N4-acetyl-N4-hydroxy-1-aminopropane	0.010	0.122	0.114	0.374	0.038	0.269	0.053	0.326	0.021	0.241
133.0375	9.4	2-hydroxysuccinamate	0.577	1.319	0.588	1.343	0.386	0.730	0.976	1.010	0.037	1.957
133.0738	16.1	N-hydroxyvaline	0.635	1.212	0.691	1.268	0.862	1.097	0.621	1.350	0.010	2.364
134.0215	8.3	3-Dehydro-L-threonate	0.590	1.432	0.130	4.219	0.494	1.631	0.494	1.471	0.079	3.753
135.0545	9.5	*Adenine	0.162	0.419	0.619	0.742	0.290	0.510	0.120	0.362	0.658	0.791
135.0545	10.0	Adenine	0.124	0.328	0.041	0.138	0.035	0.106	0.041	0.136	0.097	0.316
136.0385	10.4	*Hypoxanthine	0.635	1.187	0.008	0.300	0.007	0.288	0.004	0.217	0.489	0.809
136.0525	4.2	4-Hydroxyphenylacetaldehyde	0.480	0.804	0.395	1.383	0.825	0.933	0.734	0.895	0.651	1.131
137.084	7.7	Tyramine	0.128	1.796	0.007	3.217	0.131	2.323	0.086	2.073	0.114	1.452
138.043	8.0	*Urocanate	0.025	4.703	0.050	5.256	0.203	3.748	0.090	2.842	0.080	2.386

139.0745	8.9	L-Histidinal	0.272	0.516	0.272	0.549	0.076	0.272	0.882	0.915	0.968	1.030
140.0586	8.0	Methylimidazoleacetic acid	0.188	1.916	0.622	1.398	0.063	0.371	0.448	0.659	0.241	1.786
140.9829	13.3	Carbamoyl phosphate	0.363	11.829	0.280	33.627	0.184	42.039	0.080	12.353	0.217	4.610
140.9829	15.8	Carbamoyl phosphate	0.368	3.869	0.275	12.379	0.099	16.090	0.147	4.818	0.216	2.623
140.9829	21.6	Carbamoyl phosphate	0.108	5.341	0.094	6.723	0.018	9.973	0.104	5.267	0.047	5.467
142.0742	12.5	Ectoine	0.606	0.586	0.497	0.455	0.477	0.425	0.522	0.478	0.482	1.625
142.0743	13.3	Ectoine	0.536	0.587	0.434	0.472	0.495	0.540	0.505	0.546	0.497	1.497
142.0743	14.4	Ectoine	0.021	3.553	0.115	1.920	0.807	0.930	0.340	1.401	0.334	1.324
143.0946	10.7	Stachydrine	0.315	0.203	0.258	0.098	0.284	0.145	0.343	0.246	0.467	0.417
145.0739	8.6	[FA oxo,amino(6:0)] 3-oxo-5S-amino-hexanoic acid	0.016	0.373	0.001	0.132	0.001	0.094	0.008	0.306	0.828	0.950
145.0739	8.1	6-Amino-2-oxohexanoate	0.354	0.675	0.448	0.670	0.035	0.316	0.089	0.453	0.161	0.571
145.0739	13.6	4-Acetamidobutanoate	0.708	0.720	0.135	0.025	0.135	0.026	0.148	0.059	0.587	1.426
145.0851	15.9	4-Guanidinobutanoate	0.844	0.927	0.396	0.698	0.500	0.745	0.610	0.783	0.186	2.267
145.1102	13.9	*Acetylcholine	0.003	2.616	0.017	2.420	0.170	1.610	0.042	2.327	0.014	2.508
146.0691	16.1	*Glutamine	0.317	2.982	0.547	1.392	0.018	0.462	0.370	0.766	0.653	1.173
146.0691	11.3	Glutamine isomer	0.070	6.627	0.126	5.514	0.386	1.720	0.206	1.771	0.080	1.645
146.0692	8.0	3-Ureidoisobutyrate	0.092	3.565	0.156	7.445	0.157	6.204	0.506	1.355	0.521	1.270
146.0692	15.5	Glutamine	0.078	20.231	0.078	14.337	0.152	7.519	0.004	5.379	0.000	5.790
147.0321	6.0	Indole-5,6-quinone	0.051	3.762	0.075	4.271	0.014	3.337	0.100	2.219	0.127	1.513
147.0532	9.2	*O-Acetylserine	0.245	1.597	0.520	1.306	0.854	1.078	0.519	0.826	0.344	1.238
147.0895	9.8	N-hydroxyisoleucine	0.198	1.888	0.150	1.755	0.207	1.839	0.039	2.806	0.064	2.133
147.0895	8.5	N-hydroxyisoleucine	0.006	0.307	0.001	0.206	0.002	0.263	0.011	0.422	0.004	0.337
148.0372	8.1	D-Arabinono-1,4-lactone	0.311	0.598	0.717	0.851	0.500	0.736	0.939	0.970	0.329	1.572
148.0734	9.0	(R)-2,3-Dihydroxy-3-methylpentanoate	0.925	0.969	0.243	0.732	0.274	0.733	0.830	0.937	0.052	1.607
148.0736	7.8	3R-methyl-3,5-dihydroxy-pentanoic acid	0.663	1.255	0.483	1.466	0.706	0.826	0.547	0.722	0.348	1.795

150.0527	12.2	Ribose or isomer	0.688	0.854	0.057	0.486	0.003	0.195	0.004	0.231	0.365	0.731
150.0527	15.3	Xylose or isomer	0.410	1.785	0.558	0.732	0.055	0.293	0.096	0.397	0.364	1.376
150.0527	13.7	Arabinose or isomer	0.141	0.391	0.037	0.142	0.030	0.096	0.045	0.178	0.628	0.770
151.0633	13.4	N-Methylanthranilate	0.304	0.769	0.009	0.464	0.001	0.370	0.065	0.625	0.860	0.970
151.0633	9.3	Paracetamol	0.115	0.556	0.001	0.212	0.000	0.152	0.004	0.321	0.979	1.007
152.0473	7.8	4-Hydroxyphenylacetate	0.139	5.531	0.248	20.262	0.102	7.771	0.265	5.935	0.148	11.515
152.0684	13.3	Xylitol or isomer	0.301	1.969	0.765	1.364	0.792	1.288	0.263	0.557	0.750	0.868
153.0426	13.3	Hydroxymethylpyridinecarboxylate	0.082	49.071	0.293	50.708	0.071	283.970	0.284	591.855	0.093	119.934
153.0426	7.9	Hydroxyanthranilate	0.213	901.060	0.326	1065.093	0.262	778.568	0.148	951.672	0.150	766.549
153.0789	10.4	Dopamine	0.022	0.421	0.001	0.145	0.000	0.116	0.041	0.469	0.935	0.982
154.0266	8.2	2,5-Dihydroxybenzoate	0.085	0.180	0.046	0.033	0.045	0.031	0.050	0.054	0.780	0.838
154.0378	12.2	Imidazol-5-yl-pyruvate	0.245	1.888	0.302	0.650	0.056	0.424	0.057	0.442	0.607	1.521
155.0695	15.8	Histidine	0.218	17.100	0.259	9.043	0.370	4.893	0.986	1.006	0.115	5.046
156.0535	8.0	Imidazolonepropanoate	0.279	1.428	0.509	0.828	0.300	0.727	0.743	1.152	0.355	2.587
158.0942	4.9	oxo-octanoic acid	0.317	0.715	0.113	2.127	0.035	2.378	0.045	2.414	0.197	0.702
159.0895	13.4	3-Dehydrocarnitine	0.599	0.580	0.476	0.428	0.402	0.328	0.561	0.534	0.816	0.809
161.0477	8.6	4,8-Dihydroxyquinoline	0.407	1.413	0.207	1.510	0.763	1.099	0.577	1.171	0.919	1.042
161.0687	15.1	Aminoadipate	0.037	3.022	0.798	1.128	0.867	1.099	0.182	2.429	0.028	5.189
161.0688	9.6	O-Acetylhomoserine	0.278	2.059	0.525	1.622	0.490	1.423	0.289	1.676	0.028	2.159
161.0688	11.5	N-Methyl-L-glutamate	0.005	2.658	0.611	1.208	0.560	1.366	0.887	1.049	0.005	2.558
161.1051	13.8	*Carnitine	0.030	6.274	0.025	6.175	0.009	8.092	0.097	4.771	0.307	2.121
161.1052	12.5	Carnitine isomer	0.012	0.348	0.001	0.220	0.002	0.249	0.034	0.490	0.175	1.935
162.1003	15.2	N6-Hydroxy-L-lysine	0.085	2.061	0.007	4.250	0.022	3.611	0.048	4.617	0.774	0.898
163.0667	10.6	homomethionine	0.631	0.814	0.413	0.705	0.263	0.612	0.903	1.051	0.150	1.604
164.0685	11.5	Rhamnose or isomer	0.264	1.893	0.198	0.630	0.478	0.719	0.095	0.516	0.140	1.958
164.0686	7.9	Rhamnose or isomer	0.922	0.945	0.613	0.727	0.792	0.854	0.838	1.180	0.525	1.430
165.046	13.7	L-Methionine S-oxide	0.218	2.495	0.635	1.280	0.445	0.762	0.991	0.996	0.319	1.584

165.079	10.7	*Phenylalanine	0.038	8.696	0.141	6.321	0.237	2.429	0.069	2.000	0.113	2.620
166.049	9.9	Methylxanthine	0.372	0.715	0.021	0.324	0.012	0.264	0.182	0.582	0.110	0.565
166.0492	8.3	Methylxanthine	0.693	0.722	0.270	0.258	0.319	0.324	0.487	0.521	0.902	1.091
166.063	5.1	Phenyllactate	0.395	2.677	0.794	0.789	0.325	0.359	0.459	0.489	0.585	1.533
167.0582	11.5	Methoxyanthranilate	0.055	0.527	0.456	0.804	0.680	0.885	0.606	1.166	0.175	0.736
167.0583	5.2	Isopyridoxal	0.985	0.991	0.569	0.694	0.492	0.700	0.598	1.376	0.127	2.343
167.0583	8.0	Pyridoxal	0.007	0.420	0.002	0.296	0.001	0.220	0.490	0.757	0.082	1.936
169.0739	12.4	Noradrenaline	0.030	0.494	0.099	0.623	0.066	0.585	0.993	1.003	0.563	0.874
172.0484	9.4	Hydantoin-5-propionate	0.472	0.746	0.882	1.059	0.840	1.088	0.914	0.960	0.628	0.855
172.0484	8.0	Hydantoin-5-propionate	0.021	3.464	0.010	3.986	0.016	3.104	0.062	2.591	0.005	2.246
173.0801	13.4	Guanidinoxopentanoate	0.008	0.175	0.003	0.057	0.002	0.000	0.658	0.741	0.478	1.366
174.0641	9.4	N-Formimino-L-glutamate	0.188	2.706	0.718	1.169	0.833	1.094	0.277	1.696	0.029	2.693
174.0892	5.2	Suberic acid	0.453	0.705	0.104	0.445	0.100	0.442	0.243	0.608	0.804	0.896
175.048	8.0	aminooxohexanedioic acid	0.261	0.577	0.344	0.670	0.251	0.568	0.573	0.771	0.724	1.172
175.0633	4.6	N-Acetylindoxyl	0.336	5.600	0.389	3.940	0.197	2.928	0.345	13.593	0.401	2.048
175.0633	5.5	*Indoleacetate	0.350	4.157	0.468	2.560	0.394	1.797	0.372	5.354	0.588	1.499
175.0956	16.5	*Citrulline	0.023	2.647	0.141	2.309	0.621	1.166	0.190	1.498	0.089	1.734
179.0582	7.9	Hippurate	0.505	1.333	0.581	1.342	0.906	1.053	0.829	1.090	0.013	2.104
179.0793	17.0	Glucosamine or isomer	0.410	1.566	0.975	0.979	0.118	0.397	0.394	0.649	0.169	1.808
179.0793	15.7	Glucosamine or isomer	0.910	0.954	0.023	0.236	0.013	0.146	0.114	0.469	0.009	2.477
179.0794	11.7	Glucosamine or isomer	0.094	17.622	0.015	6.362	0.087	7.738	0.009	4.990	0.078	2.812
180.0634	15.2	Glucose or isomer	0.214	2.583	0.910	0.933	0.104	0.311	0.170	0.428	0.263	1.570
180.0898	13.9	Hydroxykynurenamine	0.018	0.457	0.003	0.310	0.002	0.296	0.064	0.549	0.208	1.341
181.0739	13.5	Hydroxyphenylpyruvate	0.034	6.924	0.067	4.412	0.129	2.404	0.036	2.271	0.028	2.524
181.0739	12.3	*Tyrosine	0.006	0.372	0.007	0.383	0.003	0.323	0.041	0.533	0.287	0.778
181.0739	11.5	3-Amino-3-(4-hydroxyphenyl)propanoate	0.028	0.459	0.016	0.403	0.005	0.301	0.044	0.510	0.296	0.772

182.058	7.8	Hydroxyphenyllactate or isomer	0.931	0.953	0.083	0.160	0.099	0.200	0.079	0.146	0.582	0.724
182.058	5.2	Hydroxyphenyllactate or isomer	0.292	0.576	0.055	0.212	0.048	0.183	0.074	0.275	0.474	0.721
182.0792	14.4	Mannitol	0.341	45.164	0.295	1.912	0.799	1.088	0.990	1.005	0.577	1.175
183.0533	8.0	4-Pyridoxate	0.027	0.466	0.001	0.292	0.001	0.284	0.037	0.493	0.231	0.734
183.0896	11.3	Adrenaline	0.031	0.409	0.001	0.141	0.001	0.102	0.017	0.354	0.727	0.912
183.0896	7.8	Normetanephrine isomer	0.038	0.565	0.020	0.496	0.004	0.348	0.054	0.552	0.419	1.612
185.1052	12.4	Ecgonine	0.003	4.144	0.495	1.218	0.109	0.660	0.763	1.148	0.022	2.301
188.1161	14.4	N2-Acetyl-L-lysine	0.017	5.761	0.028	3.506	0.094	3.559	0.385	1.444	0.076	2.484
188.1161	8.5	N6-Acetyl-L-lysine	0.410	1.746	0.226	4.018	0.531	0.788	0.193	1.807	0.496	1.281
190.0953	18.8	Diaminoheptanedioate	0.084	3.783	0.608	0.805	0.030	0.370	0.199	0.595	0.112	1.890
191.0583	7.7	5-Hydroxyindoleacetate	0.061	0.214	0.043	0.151	0.033	0.095	0.043	0.149	0.249	0.512
191.0584	5.1	5,6-Dihydroxy-3-methyl-2-oxo-1,2-dihydroquinoline	0.194	0.415	0.050	0.115	0.058	0.150	0.076	0.212	0.169	0.399
194.0425	8.5	2-Dehydro-D-gluconate	0.125	0.118	0.103	0.058	0.109	0.075	0.098	0.044	0.209	0.288
194.0426	10.1	3-Dehydro-L-gulonate	0.356	4.012	0.783	0.814	0.856	0.862	0.107	0.148	0.220	0.364
194.079	13.3	1-O-Methyl-myo-inositol	0.385	2.369	0.355	10.283	0.320	15.995	0.016	0.491	0.300	5.207
194.079	9.5	3-O-Methyl-myo-inositol	0.105	4.870	0.060	8.014	0.038	8.579	0.064	8.651	0.069	2.535
195.0531	7.8	Dopaquinone	0.314	280.389	0.338	122.268	0.324	107.048	0.262	43.774	0.111	298.712
195.0531	8.5	2-Carboxy-2,3-dihydro-5,6-dihydroxyindole	0.278	96.984	0.279	18.066	0.288	50.601	0.275	126.763	0.121	876.012
195.0757	10.2	2-Amino-4-hydroxy-6-hydroxymethyl-7,8-dihydropteridine	0.122	3.752	0.026	4.974	0.042	5.373	0.029	6.869	0.083	4.376
197.0688	7.7	N-Hydroxy-L-tyrosine	0.261	4.924	0.460	2.454	0.335	2.954	0.317	2.571	0.063	6.335
197.0688	12.7	DOPA	0.023	0.190	0.008	0.025	0.009	0.043	0.016	0.138	0.785	0.889
197.1052	8.0	Metanephrine	0.010	0.469	0.263	0.741	0.009	0.457	0.088	0.636	0.468	0.857
200.1048	5.0	[FA (10:1/2:0)] 2E-Decenedioic acid	0.407	0.727	0.076	0.465	0.104	0.478	0.439	0.756	0.140	1.532
200.1048	7.8	[FA (10:1/2:0)] 4Z-Decenedioic acid	0.643	0.856	0.219	0.636	0.231	0.609	0.760	0.883	0.021	2.037

200.1776	3.7	[FA methyl(11:0)] 10-methyl-undecanoic acid	0.551	0.500	0.737	0.719	0.812	0.802	0.730	0.703	0.498	0.427
202.1206	5.1	[FA (10:0/2:0)] Decanedioic acid	0.136	0.374	0.237	0.501	0.153	0.394	0.381	0.628	0.267	0.546
203.0794	7.9	N2-Acetyl-L-aminoadipate	0.669	0.605	0.262	0.080	0.252	0.060	0.372	3.932	0.217	3.746
203.1158	11.5	*O-Acetylcarnitine	0.073	52.011	0.120	19.002	0.045	33.596	0.002	4.976	0.176	6.821
203.1158	8.5	O-Acetylcarnitine	0.680	0.812	0.187	0.408	0.399	0.649	0.762	0.848	0.395	1.606
203.1158	8.1	O-Acetylcarnitine	0.131	0.428	0.116	0.401	0.922	0.931	0.210	0.524	0.555	0.788
204.111	18.6	N6-Acetyl-N6-hydroxy-L-lysine	0.064	3.718	0.129	3.394	0.411	1.948	0.941	0.948	0.072	4.847
205.0739	9.0	Indolelactate	0.003	0.230	0.000	0.013	0.000	0.000	0.001	0.182	0.173	0.673
205.1314	14.1	Pantothenol isomer	0.317	0.677	0.013	0.307	0.002	0.114	0.222	0.581	0.229	2.067
207.0896	7.6	N-Acetyl-D-phenylalanine	0.006	0.454	0.001	0.300	0.003	0.337	0.012	0.459	0.856	0.966
207.0896	11.2	N-Acetyl-L-phenylalanine	0.169	0.300	0.195	0.298	0.067	0.100	0.567	0.660	0.601	1.328
208.0848	15.0	Formyl-5-hydroxykynurenamine	0.129	0.464	0.025	0.202	0.026	0.210	0.521	0.715	0.170	1.931
211.048	8.0	5-(2'-Formylethyl)-4,6-dihydroxypicolinate	0.362	2.345	0.555	2.276	0.540	2.028	0.327	2.517	0.047	6.222
212.1411	3.3	[FA oxo(12:1)] 12-oxo-10E-dodecenoic acid	0.055	0.504	0.010	0.392	0.015	0.420	0.013	0.402	0.541	0.857
212.1413	3.8	[FA oxo(12:1)] 12-oxo-10E-dodecenoic acid	0.450	0.854	0.610	1.177	0.867	1.047	0.640	0.891	0.463	1.201
213.0637	7.9	N,N-Dihydroxy-L-tyrosine	0.059	0.313	0.031	0.207	0.026	0.175	0.427	4.227	0.359	3.279
214.1318	8.6	Dethiobiotin	0.096	4.427	0.156	10.913	0.038	3.369	0.079	3.275	0.076	1.666
215.0559	16.2	*Phosphoethanolamine	0.097	3.973	0.158	3.481	0.385	1.978	0.492	1.448	0.156	1.951
215.1158	5.1	2-Amino-9,10-epoxy-8-oxodecanoic acid	0.431	0.841	0.075	0.615	0.064	0.614	0.073	0.612	0.476	0.858
216.1723	3.7	12-Hydroxydodecanoic acid	0.177	1.814	0.011	4.999	0.028	6.143	0.027	5.282	0.160	1.989
217.1063	11.2	N-Acetyl-L-citrulline	0.079	3.933	0.271	6.082	0.195	2.017	0.734	1.157	0.184	1.585
217.1313	8.3	O-Propanoylcarnitine	0.306	0.590	0.258	0.547	0.174	0.466	0.479	0.719	0.632	1.270
217.1426	25.9	beta-Alanyl-L-lysine	0.084	5.993	0.304	3.061	0.934	0.961	0.607	1.465	0.961	0.981

217.1427	23.7	beta-Alanyl-L-lysine	0.094	3.902	0.232	4.856	0.951	1.022	0.337	1.734	0.436	1.286
218.1267	17.8	N2-(D-1-Carboxyethyl)-L-lysine	0.247	1.742	0.682	0.837	0.153	0.562	0.784	1.131	0.061	3.402
218.1267	13.8	N2-(D-1-Carboxyethyl)-L-lysine	0.235	0.481	0.136	0.359	0.071	0.222	0.243	0.503	0.965	1.021
219.1107	5.1	*Pantothenate	0.113	3.740	0.056	4.736	0.160	2.929	0.298	3.325	0.289	1.908
220.0847	11.4	5-Hydroxytryptophan	0.047	2.547	0.959	0.968	0.025	0.242	0.064	0.379	0.174	1.750
220.0848	8.6	5-Hydroxy-L-tryptophan	0.239	10.959	0.267	6.559	0.348	2.495	0.265	1.697	0.102	1.986
220.0849	8.0	5-Hydroxy-L-tryptophan	0.230	8.736	0.224	6.993	0.148	1.637	0.140	1.601	0.107	1.489
221.09	13.6	N-Acetyl-D-mannosamine	0.234	4.554	0.876	1.060	0.261	0.647	0.953	0.976	0.724	0.888
221.09	12.2	N-Acetyl-D-glucosamine	0.195	4.261	0.440	1.390	0.626	0.838	0.698	1.174	0.675	0.869
224.0798	8.1	3-Hydroxy-L-kynurenine	0.239	4.959	0.486	3.032	0.490	2.370	0.248	3.666	0.005	7.211
226.0953	8.2	Porphobilinogen	0.476	0.701	0.168	0.438	0.099	0.333	0.090	0.316	0.582	0.779
226.1065	12.1	Carnosine	0.036	5.863	0.253	2.820	0.507	1.313	0.344	0.706	0.223	1.367
228.0748	8.4	Deoxyuridine	0.479	0.724	0.796	1.191	0.004	0.225	0.116	0.524	0.708	1.387
230.1517	3.8	Dodecanedioic acid	0.004	0.144	0.005	0.184	0.004	0.139	0.010	0.268	0.316	0.623
231.147	8.9	O-Butanoylcarnitine	0.191	6.748	0.289	4.241	0.076	6.583	0.605	1.254	0.354	12.591
232.1059	9.4	N6-Acetyl-LL-2,6-diaminoheptanedioate	0.022	6.557	0.303	5.125	0.213	3.713	0.979	1.009	0.132	2.595
232.1059	15.5	N2-Succinyl-L-ornithine	0.185	33.008	0.006	0.017	0.013	0.126	0.766	1.390	0.079	16.161
232.1212	9.1	Melatonin	0.225	7.174	0.612	0.623	0.717	1.567	0.654	1.555	0.385	2.195
236.0797	12.4	L-Formylkynurenine	0.006	5.766	0.010	4.817	0.100	2.414	0.214	1.906	0.009	3.924
240.122	13.2	Homocarnosine	0.207	0.572	0.702	0.840	0.001	0.258	0.619	0.833	0.259	1.736
240.1222	16.9	Homocarnosine	0.547	2.117	0.057	0.042	0.055	0.030	0.108	0.198	0.640	1.369
240.1222	14.1	beta-Alanyl-N(pi)-methyl-L-histidine	0.264	0.618	0.011	0.361	0.010	0.353	0.265	0.694	0.206	1.830
240.1725	3.1	oxoTetradecenoic acid	0.021	0.379	0.003	0.168	0.003	0.131	0.006	0.225	0.592	0.860
241.1175	16.7	Tetrahydrobiopterin	0.069	0.220	0.060	0.190	0.054	0.166	0.559	2.107	0.744	1.204
242.0904	7.7	Thymidine	0.168	0.528	0.696	1.301	0.038	0.323	0.203	0.528	0.841	1.140
243.0856	12.4	Cytidine	0.339	0.563	0.231	0.472	0.056	0.200	0.064	0.222	0.182	0.442

244.0694	12.2	Pseudouridine	0.240	1.963	0.380	0.685	0.039	0.377	0.037	0.386	0.732	1.270
244.0694	10.1	Uridine	0.492	0.791	0.243	1.828	0.433	0.769	0.554	0.817	0.989	0.997
245.1489	26.6	beta-Alanyl-L-arginine	0.037	13.169	0.106	6.450	0.141	2.238	0.133	3.359	0.010	4.960
248.116	11.9	6-Hydroxymelatonin	0.029	8.813	0.227	3.635	0.341	1.989	0.663	1.372	0.040	3.219

Table S2 Sphingosine metabolism

Mass	RT	Putative metabolite	P value C/A	A/C	P BC value	B/C	P value CC	C/C	p VALUE CD	D/C	p Value EC	E/C
295.2512	4.4	Sphingatrienine	0.985	1.006	0.286	0.737	0.850	0.947	0.670	0.881	0.628	1.123
315.2774	3.6	Dehydrophytosphingosine	0.391	1.507	0.798	1.108	0.460	1.360	0.536	1.294	0.016	2.881
315.2774	4.2	Hydroxysphingenine	0.881	1.171	0.406	0.222	0.486	0.349	0.428	0.258	0.494	2.300
327.3137	4.2	N,N-Dimethylsphing-4-enine	0.006	6.526	0.013	23.310	0.007	27.210	0.038	18.263	0.020	3.368
465.3454	3.8	LysoSM(18:1)	0.756	0.901	0.931	0.976	0.669	1.124	0.918	0.968	0.419	0.792
467.3612	3.8	Sphinganinephosphocholine	0.195	0.569	0.375	0.719	0.933	1.034	0.637	0.840	0.075	0.472
481.4494	4.1	Dodecanoylsphingenine	0.024	ND	0.040	ND	0.025	ND	0.243	ND	0.017	ND
509.4814	4.1	Tetradecanoylsphingenine	0.008	8.294	0.021	6.755	0.022	6.433	0.220	3.380	0.002	5.397
537.512	4.1	Hexadecanoylsphingenine	0.005	20.235	0.028	12.052	0.047	6.116	0.141	3.591	0.000	8.375
555.5225	4.1	hydroxyhexadecanoylsphingenine	0.570	1.284	0.028	2.729	0.037	2.700	0.164	2.205	0.935	1.030
563.5277	4.0	Octadecenoylsphingenine	0.003	11.861	0.030	7.152	0.054	4.992	0.320	3.699	0.001	6.616
565.5436	4.0	Octadecanoylsphingenine	0.026	3.912	0.214	1.581	0.704	1.133	0.880	1.066	0.030	4.746
569.538	4.1	Cer(d18:0/h17:0)	0.373	0.658	0.259	0.604	0.299	0.635	0.964	1.020	0.663	1.169
647.6217	4.1	Tetracosenoylsphingenine	0.002	27.212	0.027	10.818	0.024	5.612	0.123	3.305	0.002	19.136
702.5675	4.4	Hexadecanoylsphingeninephosphocholine	0.002	13.250	0.031	5.520	0.052	4.231	0.391	1.789	0.002	8.834
812.677	4.3	SM(d18:1/24:1(15Z))	0.006	20.757	0.030	7.319	0.066	5.979	0.432	2.195	0.004	14.297

Table S3 Calprotectin values. ND = not determined

Subject	Calprotectin wet	Calprotectin dry
PA01	2272.3	15056
PA03	1130.4	6161
PA04	2438.7	8088
PA05	2581.7	7876
PA06	2076.3	6022
PA07	2102.2	5751
PA08	2187.9	9334
PA09	2262.3	7371
PA11	3114.22	19147
PB01	1841.6	6335
PB02	2390.1	19899
PB03	47.7	211
PB04	2221.6	7056
PB05	2341.1	7695
PB06	1704.8	6785
PB07	1999.9	6760
PB08	2056.2	9720
PB09	1808	7659
PB11	2216.346	7803
PC01	1673.7	6676
PC02	2324.6	20520
PC03	5.8	29
PC04	2000.5	5878
PC05	2394.3	7967
PC06	1459.5	3909
PC07	1535.8	4985
PC08	2076.7	9321
PC09	1797.6	8315
PC10	296.393	713
PC11	1803.814	7743
PD01	1685.8	7400
PD02	2563.7	20312
PD03	88.4	373
PD04	1723.5	6171
PD05	2460.9	8214
PD06	39.1	139
PD07	718.1	2338
PD08	2298.3	7376

PD09	2055.8	11449
PD10	77.121	183
PD11	106.118	444
PE01	2085.3	6701
PE02	1052.7	4221
PE03	2054.6	12284
PE04	2327.7	9084
PE05	2495	7276
PE06	1712.1	4816
PE07	1632.2	7821
PE08	2169.5	7948
PE09	2470.2	11241
PE10	2418.17	6754
PE11	2355.175	9293
HC01	ND	ND
HC02	8	25
HC03	101.3	300
HC05	3.7	11
HC06	ND	ND
HC07	ND	ND
HC08	8.5	20
HC10	5.7	19
HC11	8.3	23

Characterisation of unknown markers

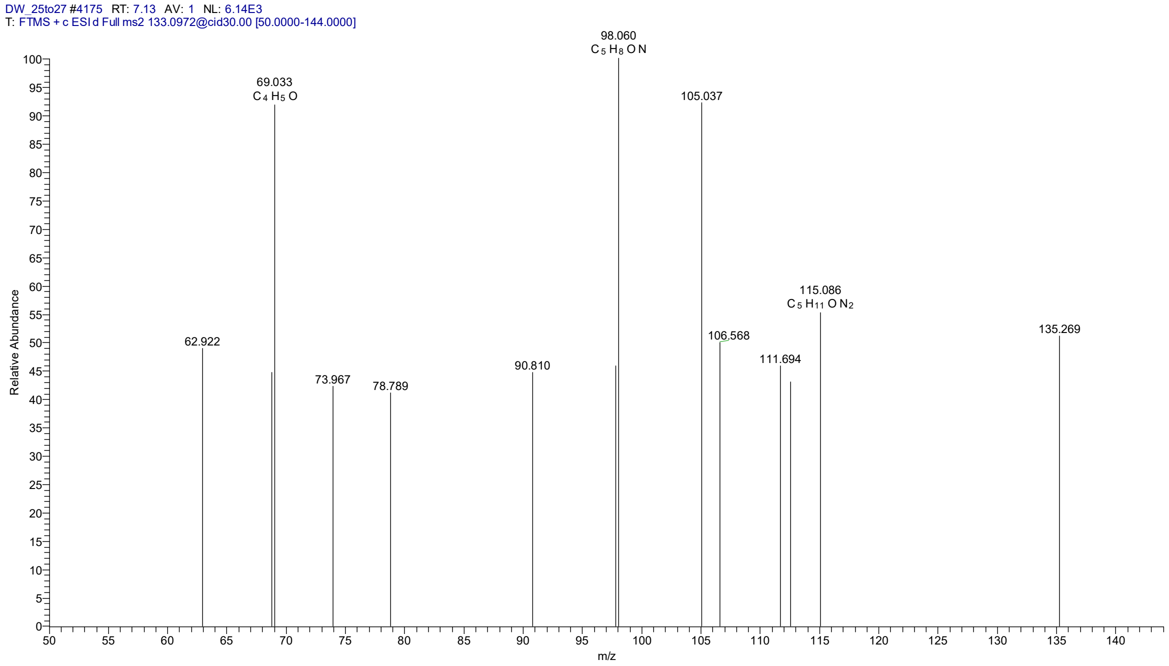

Figure S4 MS^2 fragments of ornithine isomer.

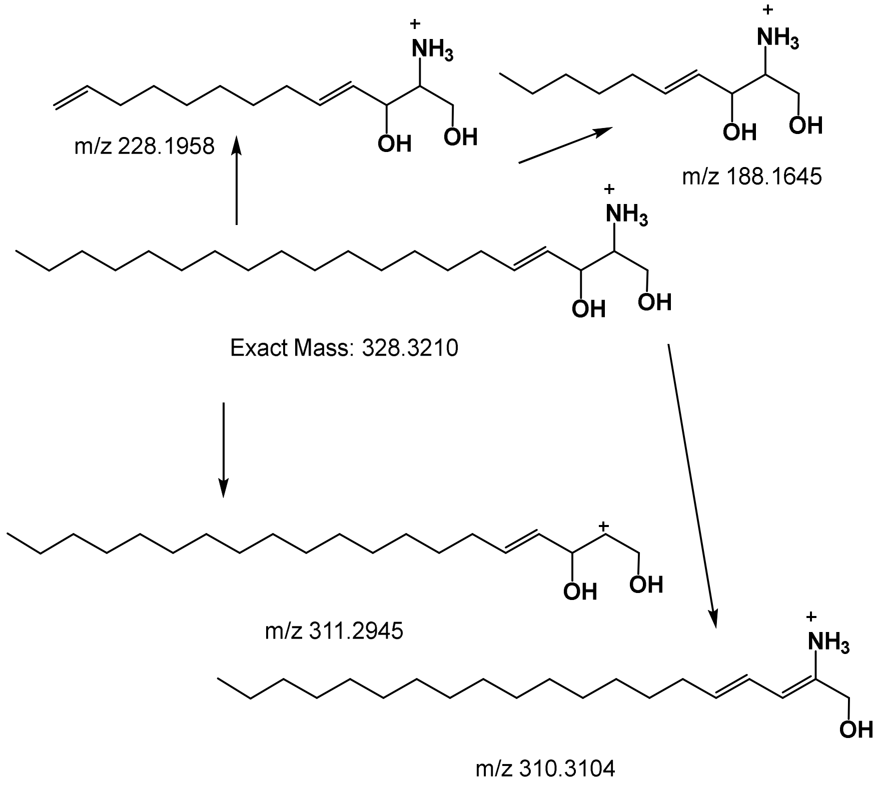

Figure S6 Proposed fragmentation of C20 sphinganine.

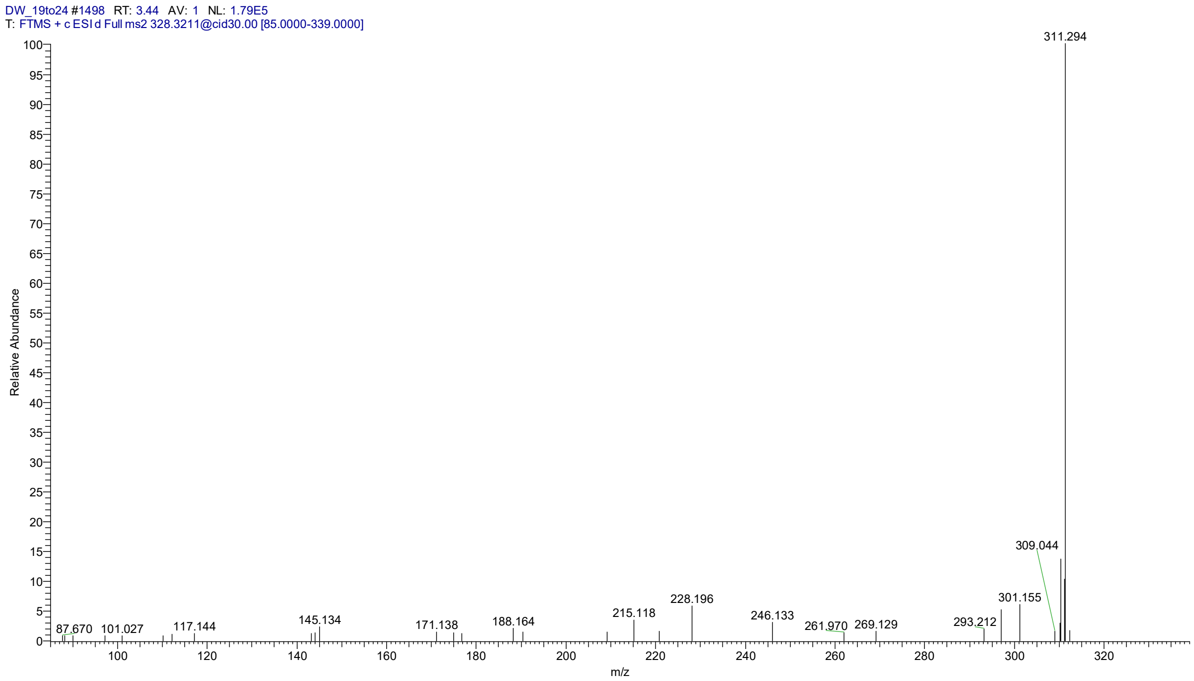

Figure S7 MS² spectrum of C20 sphinganine.

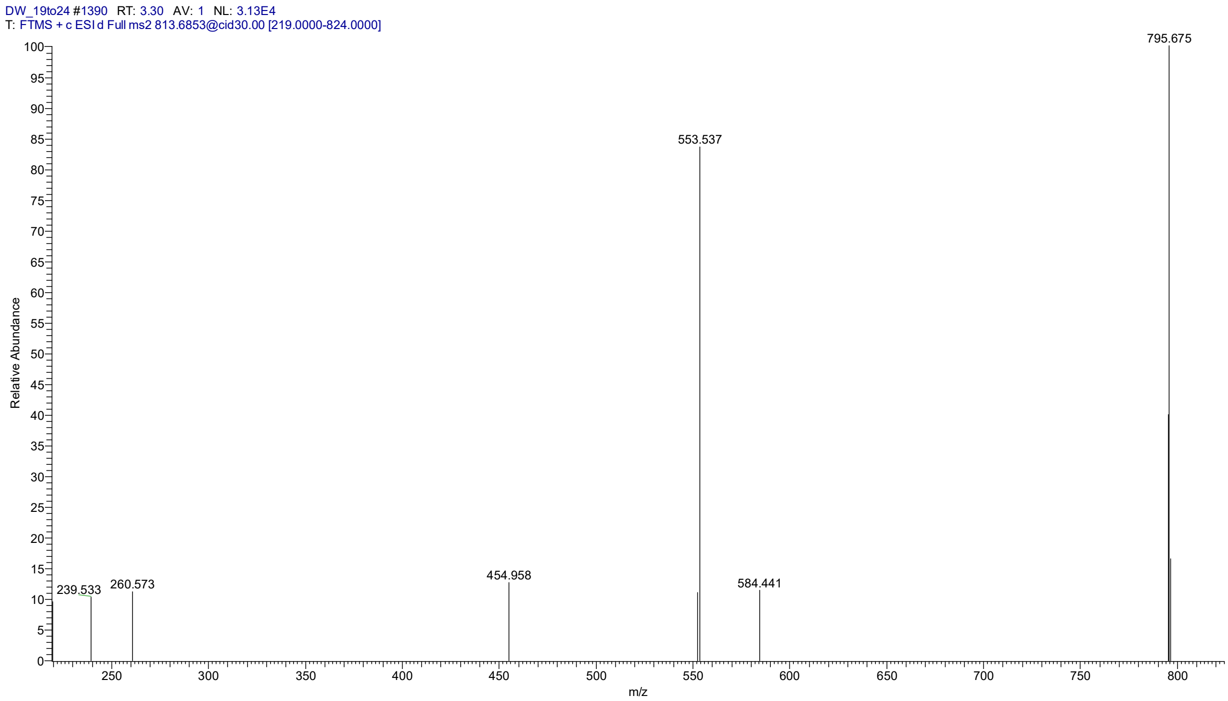

Figure S8 MS² spectrum of Ceramide d18:1 24:1.

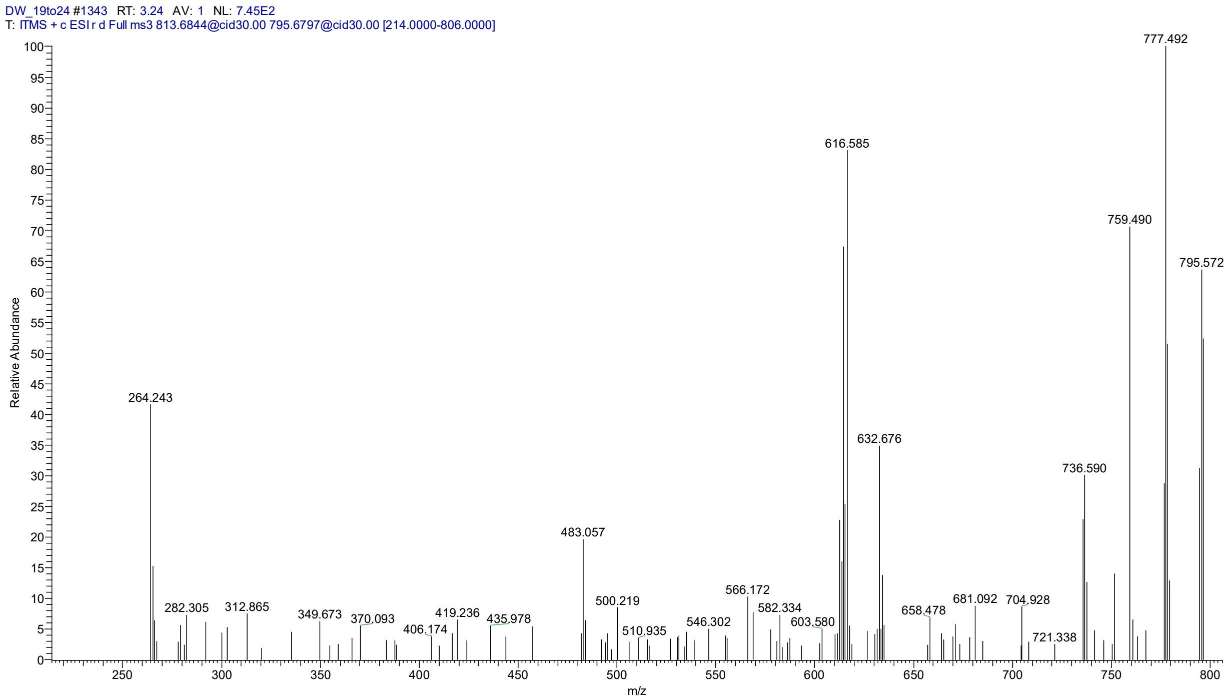

Figure S9 MS[3] spectrum of Ceramide d18:1 24:1 (795.5 ion).

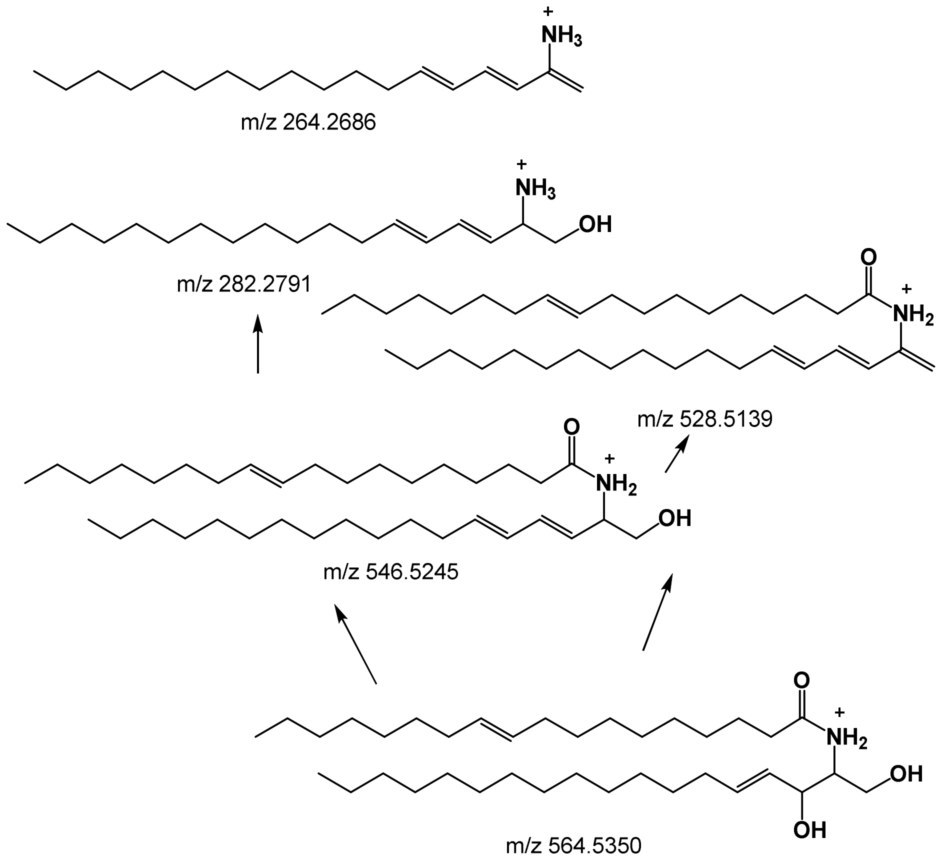

Figure S10 Proposed fragmentation of octadecenoylsphingenine (MS[2] spectrum shown in figure S11)

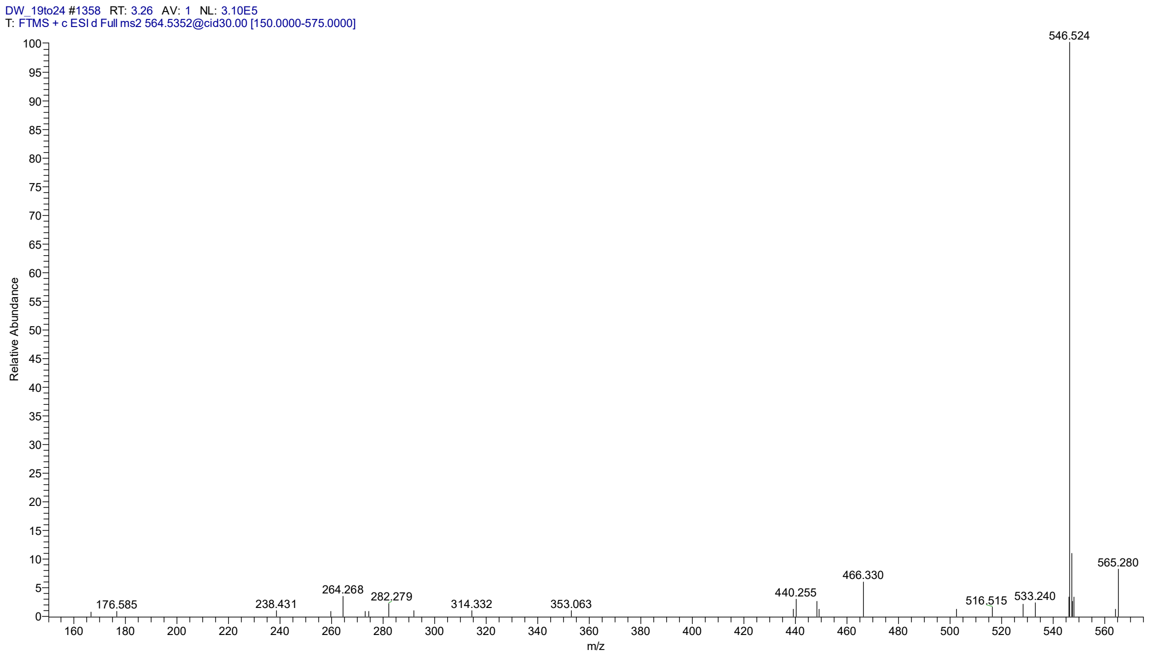

Figure S11 MS^2 spectrum of octadecyl sphinganine.
